# Resource-efficient data transmission for WiFi-capable bio-loggers based on machine learning

**DOI:** 10.1371/journal.pone.0354146

**Published:** 2026-07-24

**Authors:** Wilhelm Kerle-Malcharek, Karsten Klein, Martin Wikelski, Falk Schreiber, Timm A. Wild

**Affiliations:** 1 Department of Computer and Information Science, University of Konstanz, Konstanz, Germany; 2 Department of Migration, Max Planck Institute of Animal Behavior, Radolfzell, Germany; 3 Department of Biology, University of Konstanz, Konstanz, Germany; 4 Faculty of Information Technology, Monash University, Clayton, Australia; Xidian University, CHINA

## Abstract

Bio-logging is a popular method for data collection in animal research, especially for hard-to-observe animals. Newer bio-logger generations utilise WiFi technology, enabling researchers to collect high-resolution data at the cost of higher energy expenditure of the devices. In this study, we elaborate on how state-of-the-art loggers can benefit from even the simplest methods to reduce transmission costs. We employ machine learning techniques, specifically small decision trees, to enable a bio-logger to recognise a chosen behaviour based on its sensor readings. Based on the recognised behaviour, the logger filters which data to transmit, reducing transmission time and, thus, the logger’s overall energy consumption. Using a controlled dataset, we exemplify the training and evaluation of such decision trees. Using those, we evaluate the reduction of energy consumption based on a state-of-the-art bio-logger, the WildFi tag. We demonstrate that for WiFi-enabled bio-loggers, decision trees are highly beneficial when used as a data filter. We illustrate that filtering with decision trees yields energy savings of 14.68% in realistic scenarios for transmitting data. We provide a full pipeline from data collection to deployable software to holistically elaborate on how to use off-the-shelf solutions to achieve practical gains for animal behaviour. Our results suggest that decision trees can be an effective tool for enabling bio-loggers to detect specific behaviours. Lastly, we emphasise that our approach highly benefits from the use of gyroscopes, a sensor type that mostly sees use for off-board instead of on-board labour. We contribute an investigation of energy consumption reduction of WiFi-enabled bio-loggers through the utilisation of controlled data transmission using machine learning. We offer a promising pathway for enhancing the longevity of such a state-of-the-art bio-logger, maintaining WiFi benefits. Ultimately, we support more efficient and, thus, more sustainable wildlife monitoring practices on the example of the WildFi tag.

## Introduction

The study of animal behaviour has a rich history, driven by the interest in understanding the mechanisms and reasons behind an animal’s decision making [1–4]. Advances in technology have significantly contributed to corresponding research, allowing for detailed data collection. For instance, measuring the diving capacities of Weddell seals [5], GPS tracking of birds and wolves [6–8], and tracking the acceleration patterns of fish and other animals over time [9–12] to infer knowledge about their behaviour are possible nowadays. The animal-borne electronic recording devices that enable such works are called bio-loggers [13]. Bio-loggers tremendously increased the understanding of animal behaviour, fueling the interest in further advancing bio-logging technologies.

In particular, inertial measurement units (IMUs), which include 3-axis accelerometers, magnetometers, and or gyroscopes, have become essential in studying animal movements [14–18]. State-of-the-art bio-loggers, such as the LoRaWAN bio-logger [19] and the WildFi tag [20], integrate such IMUs with GPS and other sensors to gather information-rich datasets. Naturally, more sensors that capture information with increasing resolution (more data points) also put a higher strain on the battery lifetime (more measurements require more energy) of bio-loggers, as well as their storage capacities (more readings produce larger file sizes). Since the recorded information becomes denser, novel data transmission approaches gained popularity and advancements to circumvent the higher storage demands by simply freeing up storage of data that has already been transmitted [20–25]. The downside to data transmission is that it is one of the most energy-consuming activities of distributed embedded systems [26]. Thus, while WiFi technology, as it is used by the WildFi tag, allows for a transmission speed of about 230kB/s, the bio-logger runtime is shortened compared to just storing recorded data. For comparison: A WildFi tag measuring with a 9-axes IMU and transmitting its data via WiFi has a reduced runtime of approximately 6 hours [20]. Still, using WiFi protocols is an advancement, essentially solving storage limitations, allowing retrieval of data without the need to recapture the animal, and gathering fine-grained information, leveraging the systems to be capable of handling big data. These challenges and possibilities necessitate a trade-off between data resolution and device operating time, especially since bio-loggers must be small and unobtrusive to minimise disruption to the animals. This is why they typically have limited energy storage, constraining data resolution or shortening operating time even further.

The Internet of Things (IoT), which concerns itself with networks of interconnected devices that communicate and exchange data, offers valuable strategies for managing energy consumption for those interconnected devices through software and hardware solutions [27–29]. By employing sensor fusion, IoT systems can deduce specific conditions of tracked objects [30] and optimise sensor usage and power management accordingly [27]. This concept of smart energy management is particularly crucial for bio-loggers, as it offers ways to tackle the trade-off between data resolution and energy consumption.

Recent publications suggest that machine learning can be used to detect specific states of animals [31]. Generally, machine learning on animal behaviour time series data to classify their behaviour at a certain point in time has seen several approaches and is considered to hold a lot of potential [32,33]. Bidder et al. used the k-nearest-neighbour algorithm to provide easy-to-use solutions for non-specialists in machine learning [34]. Chakravarty et al. present several approaches towards optimisations for behaviour recognition using accelerometers, like the use of high-fidelity data [18] or even looking at it at a biochemical level [17]. As the topic unfolds for bio-loggers, different methodologies are being discussed and applied for them, as well [35,36]. Given the potential of recognising a specific behaviour of an animal, researchers like Korpela et al. even transferred parts of the machine learning approaches onto the bio-logger and utilised it. They explored the idea of on-board classification for preserving energy by limiting the recording of data to behaviours of interest. They trained decision trees with the criterion that they exhibit a good balance of cost to accuracy and based their data on 3-axis accelerometers [37]. With our study, we want to further this thought. We exemplarily show that onboard pattern recognition works and is facilitated through sensor fusion by integrating sensors other than accelerometers, like gyroscopes, which are often neglected for such purposes, with them mostly playing a secondary role in classification tasks [38]. We put into perspective the net costs of on-board classification to show that the on-board recognition is feasible, too. We offer comprehensive insights into how simple off-the-shelf machine learning can be used to improve not only data sampling but also data transmission strategies for state-of-the-art bio-loggers.

To achieve this, we created a small, controlled dataset that enables us to determine accruing energy costs along the processing chain and to explore the importance of different sensors for onboard classification. As animal behaviour comes with unpredictable difficulties and we want to determine future potentials in a controlled setup, we created this dataset with 3 humans who wore those biologgers on their body. Through our methodology, we complement the work of Korpela et al. by incorporating more sensor types, elaborating on the effective theoretical energy savings with a focus on data transmission, and elaborate how to translate this into the actual data transmission in the field. We argue that the overall gain in runtime of a bio-logger can be significantly improved.

## Materials and methods

The general methodology we follow to reduce the energy consumption of bio-loggers is based on the premise that we want to reduce the time spent and the length of messages transmitted. To achieve this, we make use of pattern recognition with machine learning, leaning into the emerging field of TinyML. We train a model to distinguish different animal behaviours based on sensor data, try to reduce the required data for recognition, and supply the bio-logger with the finished model. With the help of the classifier, the device makes decisions based on the recognised behaviours about which data to store and transmit, thus filtering the transmitted data.

### Hardware

**Fig 1:**
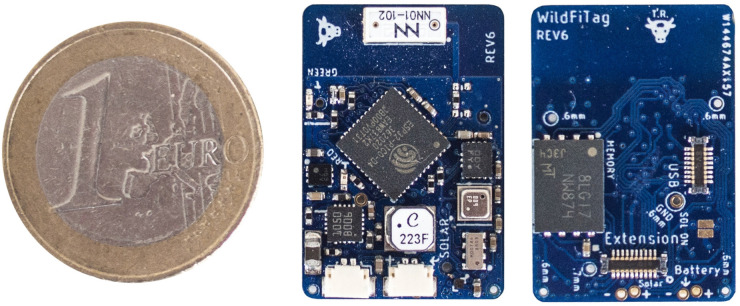
WildFi tag illustration. An illustration of the WildFi tag from the github page [39] of the original publication [20]. On the left is a coin to give a perspective on the device’s size, and next to it is the main device from front and back. Republished under a CC BY license, with permission from Timm A. Wild, original copyright 2022.

The bio-logger we use is the WildFi tag [20] (Fig 1). The WildFi tag is a cutting-edge bio-logger utilising advances in the fields of IoT and bio-logging alike. The size of the logger is 25.95mm×17.85mm×0.6mm, and its weight is about 1.28g without the GPS extension, which makes it small and light enough to be used for animal behaviour purposes, even on small animals. The device runs with an ESP32 Pico D4 microcontroller unit whose main CPU has a 240MHz maximum clock speed, 4MB flash memory, and 520kB RAM.

The WildFi can store up to 256MB of sensor readings in its NAND flash memory. It is equipped with a Bosch BMX160 [40] 9-axis inertial measurement unit (IMU), thus including a 3-axis accelerometer, gyroscope, and magnetometer, allowing for the exploration of the impact of sensor fusion for our purposes. The recording frequency of the IMU is 50 Hz, and the data it records lies at 900B/s. It also has a natively integrated environmental sensor, the Bosch BME680 [41], with a sampling frequency of 1 Hz and a recording rate of 10B/s. The WiFi transmission rate of the WildFi lies at 230kB/s with an average current consumption of 108*mA*, as measured by the authors [20]. The antenna used for transmission is the NN01–102 antenna [42].

Since the purpose of this work is to save energy on the bio-logger, we only executed the data acquisition and the final classification model on the bio-logger. We outsourced the modelling process to a tower PC. The PC used for the training had an 11th Gen Intel(R) Core(TM) i7-11850H 64-bit processor, 2.5GHz dual-core, and 32GB RAM. We used Python 3 and the SKlearn library [43] for generating our machine-learning models.

### General concepts

Commonly, energy consumption can be described as the product of the required power in watts multiplied by the time the system is running. It is constituted by E=P·t or directly through the respective units J=W·s. Therefore, to reduce energy consumption, we can reduce either the wattage required for an action or the time *t* invested in that action. We focus on reducing *t* by reducing the to*t*al time required for data transmission, as we can control *t*his aspect more easily with software solutions. The time required for a transmission *T* can be calculated with $T=\frac{L}{R}$, with *L* being the length of a message and *R* being the transmission rate of the transceiver. The calculation is simplified to obtain a clearer idea of the impact of our methodologies, albeit various factors influence the effective transmission time and the overall consumed energy. For one, the nominal data transmission rate of R=230kB/s can be influenced by various factors. One example is retransmissions that occur when transmitted packets are erroneous, where the effective rate is proportional to how much time it takes to supplement the missing data packets, thus prolonging transmission time. Signal strength and interference are slowing down the transfer speed as well, which is likely to happen in the field through vegetation, weather conditions or other factors. Another factor of influence is the overhead created by establishing a WiFi connection to transmit data. It has to scan for valid channels, establish a connection, and only then can it start transmitting the data. The NN01–102, used by the WildFi, has a bandwidth of 2.4GHz, which translates to up to 14 channels to be scanned, with a scan time of 120ms each [44]. Therefore, searching for a channel can take 1.68 seconds. On top of that, there is the overhead for authentication, 4-way handshake, and DHCP, if required [44]. The time this takes, again, depends on the connection quality and can range from a few milliseconds up to the timeout configuration. Due to this dependency on environmental conditions and settings, we assume a range between 0s (representing an ideal scenario with negligible overhead) and 1s (as an upper bound for estimates, which can actually be much higher) for connection establishment. Considering the scanning times, this leaves us with a roughly estimated connection time between 120ms and 2.68s. The range of current while trying to connect lies between 95-240mA [45], depending on the configuration. Considering that the supply voltage of the WildFi is specified to be *U* = 3.75*V*, we can deduce an estimate for the overall energy cost of the WildFi connection to be in the range of $E_{lower}=3.75V\cdot 0.095A\cdot 0.120s=0.04275J$ and $E_{upper}=3.75V\cdot 0.240A\cdot 2.68s=2.412J$. On average, we can assume $E_{c}\approx 1.2J$ of extra costs per established WiFi connection without heavy optimisation. Assuming a strategy, where the WildFi transmits information in bursts, we can assume the full energy costs of a tag over its lifetime to be described by:


$$\begin{array}{c} E_{tx,total}=\sum_{i=1}^{n}E_{c}+\frac{P\cdot L_{i}}{R} \end{array}$$ (1)

where n describes the number of bursts, $E_{c}$ is the energy consumption for a single connection establishment, and $E_{tx,total}$ is the overall energy expenditure for transmissions over time. Since connection overheads are expensive, the WildFi employs batched data transmission, has specified WiFi channels to avoid scanning through gateways and, furthermore, utilises duty cycles and further ways of optimisation to account for that. Because of the interplay between environmental circumstances and the optimisations of the WildFi, for simplicity, for the remainder of this work, we will consider the following assumptions based on the original WildFi publication: Firstly, we assume an invariant data transmission rate R=230kB/s. Secondly, we neglect overheads for transmission time, as they are included in the energy costs the original authors show, for the calculations of transmission costs. Also, we will assume a supply voltage of *U* = 3.75*V*. Lastly, we additionally assume that *T* = *t*, meaning that the time over which energy is consumed directly corresponds to the transmission time. To calculate the energy consump*t*ion, we derive P=3.75V·0.108A=0.405W, which leaves us with the following equation to calculate energy expenditure for a single data transmission for the WildFi tag:


$$\begin{array}{c} E_{tx}=\frac{0.405W\cdot L}{2.3\cdot 10^{5}B/s} \end{array}$$ (2)

**Fig 2:**
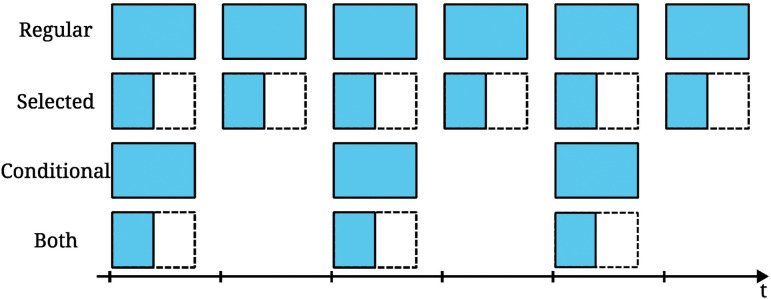
Energy saving concept illustration. This image illustrates the concepts behind the reduction of transmission time with the help of contextual transmission. “Regular” means to send every message whole. “Selected” means to send only a selection of information. “Conditional” means to send messages when a condition is met. “Both” corresponds to a combination of conditional and selected data transmission. Each blue box indicates a message that is being sent. The dotted lines illustrate that a portion of a message is not transmitted. The bottom arc shows a progression over time, without a specified unit of time, to allow for an abstract comparability between the different approaches.

As a result, in our setup, the reduction of energy expenditure directly corresponds to reducing *L*. To reduce the time, there are multiple options as Fig 2 illustrates.

Given a set of messages with a uniform length *L* per message, reducing the overall time of transmission can be achieved by either reducing the number of messages sent, reducing *L* of each message, or both. A reduction of the number of transmitted messages can be achieved through conditional data transmission, where a bio-logger sends data only when specific patterns are detected. This approach minimises network traffic while preserving the integrity of each message and relies on recognising patterns we will refer to as behaviours of animals. For a bio-logger that can detect a behaviour *B* with 100% accuracy, data transmission can be limited to occurrences of *B*, reducing the proportion of messages proportionally to its frequency. Alternatively, data can be transmitted when *B* is not detected, though this risks omitting relevant information if behaviours overlap. Ideally, behaviours of interest and *B* should be mutually exclusive in this scenario. In practice, perfect detection is rare. Therefore, the goal is to minimise the loss of relevant data. For instance, if a classifier misclassifies 1% of the time, and the target behaviour is common, such as when an animal is sleeping, missing a few instances may be acceptable.

If maintaining the density of data points is essential, reducing the message length *L* is an alternative. This reduction can be achieved through selected data transmission, where decisions are made about which values, such as sensor data or their encodings, to include in each message. This process involves either encoding or compression techniques, as discussed in prior research [46,47]. While encoding preserves information, data compression may introduce some distortion. Both methods aim to reduce message size while retaining the core information.

Generally, each data transmission protocol benefits from the reduction of data to transmit if preserving energy is the goal. Different data transmission protocols have their respective limitations and advantages, however. While the SigFox protocol allows only about 140 messages with 12byte each, its transmission range can reach distances of several kilometres [25]. On the other hand, the WiFi protocol of the WildFi tag can transmit vastly more data than the SigFox, with a transmission range of only a few hundred meters. In this study, we consider the WiFi protocol of the WildFi tag and an example scenario where the transmitted data are the features required for behaviour detection. These features might include direct sensor values or proxy methods from literature, such as VeDBA [48,49], which encodes all three acceleration axes into a single value correlating with energy expenditure. Such approaches preserve key information while reducing the size of the data transmitted from the bio-logger to the receiver.

### Pattern recognition procedure

**Fig 3:**
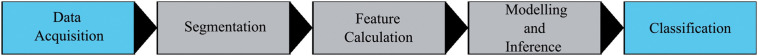
Activity recognition chain. This is a modified version of the activity recognition chain pipeline by Bulling et al. [50]. From left to right, the single boxes show the abstract steps that are appropriate to achieve a successful classification. We additionally marked which step happens on which type of device, either PC or bio-logger, by colouring the PC steps grey and the onboard steps blue. The original author permitted us to use their graphic.

To produce a model, we utilised the framework for effective pattern recognition from recording sensor data to executing a classification called the “activity recognition chain” (ARC) by Bulling et al. [50]. We applied it as the frame of our workflow to establish a well-accepted starting point. As the simplified version of that ARC in Fig 3 shows, there are 5 main processing steps.

#### Data acquisition.

The data acquisition corresponds to the actual measuring process executed by sensors. These sensors can be anything from acceleration sensors to GPS or humidity sensors. We acquired our data by recording it using the WildFi tag. The activated sensors for measuring were the IMU with accelerometer, magnetometer, and gyroscope, as well as the environmental, and the GPS sensor. The latter was attached and active for technical reasons, but was not considered in further processing. In general, the logger was configured to read 50 Hz IMU and 1 Hz GPS data. The recorded raw data was then decoded and converted into the CSV file format for further processing. The resulting file represents a time series where each row is a timestep. All timesteps are 1 second apart, and each row is an n-tuple with every measurement from its respective moment, including the timestamp and 50 values per axis of the accelerometer, the gyroscope, and the magnetometer. When we refer to a data point, we refer to one of the n-tuples.

#### Segmentation.

The segmentation step is the data preprocessing step. For this step, we removed any entailing readings that did not belong to our test case and labelled the remaining data afterwards. Since the IMU samples with 50 Hz bursts per sensor, we took the average of all 50 readings per second as the corresponding value for the respective sensor. For tracking animal behaviour, this is likely to smooth out information that is crucial to detect complex movement patterns, but for our experimental data, this approach sufficed.

#### Feature calculation.

Feature calculation is a preparatory step to provide features that are capable of distinctively describing the target behaviour. The question of which features in the form of sensor values are especially descriptive of a given behaviour of an animal proves particularly difficult. Too many features for training will result in high execution times for the modelling. Too few might not capture an animal’s behaviour sufficiently.

Table 1 shows a small derivative version of Table 1 from the work of Williams et al. [51], who put into perspective different types of sensors and in which context they suit best. We incorporated this part of their work as part of our feature calculation step since it helps to rule out potentially irrelevant sensors, given knowledge of the context of the usage of the bio-logger. Various analysis techniques can and should be utilised to filter features further if the actual feature space amounts to an unfeasibly long time for modelling.

**Table 1:** Mapping from sensor types to use cases.

Sensor type	Examples	Description	Relevant Questions
Location	GPS	Track positions	Space use; interactions
Intrinsic	Accelerometer; Gyroscope; Magnetometer	Patterns in body posture	Behavioural identification
Environment	Temperature	External conditions	External factors

A reduced version of Table 1 from the work of Williams et al. [51], where the authors break down in which cases which sensors are appropriate. The “Sensor Type” column shows the possible contexts of sensors. The column “Examples” covers which sensors are fit for the sensor types. The “Description” column indicates the kind of information that can be expected, and “Relevant Questions” hints at what research questions can be addressed with the respective sensors.

As we pursued the goal of putting the abilities of sensors into perspective, we limited our relevant sensor types to intrinsic ones, namely the accelerometer and the gyroscope, omitting the magnetometer for simplicity. Thus, our feature space is limited to the three axes of the accelerometer (AX, AY, AZ) and the gyroscope (GX, GY, GZ), amounting to a total of 6 input features.

#### Modelling and inference.

The Modelling and Inference step describes the procedure of the actual training of the model. There are various methods for so-called TinyML, machine learning on microcontrollers [52], which can require as little as 16kB RAM, like TensorFlow Lite by Tensorflow [53], which easily fits into the WildFis memory. The TinyML method we used was decision trees [54] due to their hierarchical structure, which helps identify the most impactful features for describing behaviour and their predictable number of calculations per classification. Unlike other machine learning approaches, decision trees require only a few hyperparameters to be tuned before the actual training. The primary parameter in our case is *k*, which sets the maximum tree depth, allowing us to limit the computational steps during the classification process on the bio-logger and, thus, actively influence the current consumption during the process. However, we outsource the training process to a PC to preserve energy.

In machine learning, the term hyperparameter tuning refers to the act of adapting the hyperparameters and retraining the model with the new hyperparameter to evaluate the quality of the different models and pick the hyperparameters that return the model that fulfils the desired qualities the best. After a few iterations with our data, we obtained *k* = 7 as a well-performing tree depth. We also worked with *k* = 14 according to a related project preceding this study, where this was found to be the maximum depth for optimal usage of the WildFi tag. As for the models, we pursued two different directions. The first direction was to use all features for a decision tree, whose purpose is to investigate the overall functionality of decision trees for behaviour detection, as well as the functionality of the deployed code. We trained the model with all three axes of the accelerometer and the gyroscope and deployed the resulting decision tree on the WildFi tag.

The second direction introduces the selected data transmission. We applied the hyperparameter tuning approach to the feature space such that we obtained a model for each permutation of the desired training features. We checked how well the decision trees perform in terms of F1 score, the harmonic mean between precision and recall of a classifier, and accuracy for each of the resulting models. For the testing, we split the datasets into training and testing data with a 0.3 ratio to prevent data leakage. Afterwards, we compared the different scores and were able to pick a feature setup that can be considered good enough for the recognition of a chosen behaviour. In the end, we had two decision trees, one with all and one with a subset of features.

#### Classification.

The classification of real-time readings of data happens on the bio-logger, as this is where we want to achieve energy savings. For this, a working classifier has to be deployed on said bio-logger. We implemented a small Python program, which produces the decision trees with the help of the recorded data and outputs them as header files, which can be included by the WildFi. With a small modification of the logger’s firmware, it is then able to use the produced tree. However, the on-board classification introduces additional calculations required for the preparation of classification or the classification itself, which are not allowed to be more expensive than the savings achieved. To estimate the costs of an operation, we assume that the WildFi runs at its most power-consuming mode with a 240MHz clock speed. According to its datasheet, the ESP32, at 240 MHz, requires about one clock cycle for a simple operation like addition or comparison, which lies at $\frac{1}{240\times 10^{6}}Hz=4.17ns$. Say we apply an operation with 100 such calculations on a sensor reading, such that an entire operation takes 417 *ns*. Assuming 10 sensors, this ends in on average 4170 *ns* per sampling. The actual consumption can be roughly calculated with $P=C\times V^{2}\times f$ [55], where C is the capacitance, *U* = 3.75*V*, and *f* is the clock frequency. Since C varies, we use active power consumption as a reference. The active current is, therefore, P=0.24A·3.75V=0.9W. Thus, with $E=P\cdot t=0.9W\cdot 4170\cdot 10^{-9}s=3753\cdot 10^{-9}J=3.753\cdot 10^{-6}J$. Now, say we transmit the smallest information we can with only 2 bytes per sensor per second with the assumed 10 sensors, thus 20B/s. We can calculate the energy costs with $E=\frac{0.405W\cdot L}{2.3\cdot 10^{5}B/s}=\frac{0.405W\cdot 20}{2.3\cdot 10^{5}B/s}=3.52\cdot 10^{-5}J$, which is an order of magnitude more than the calculations done beforehand, even in a scenario, where the transmitted information would be small. If we use a more expensive operation, like division with approximately 32 clock cycles, we arrive at $\approx 1.2\cdot 10^{-5}J$, which is still almost a third of the cost of transmission. Therefore, as long as the on-board calculations are not extremely extensive, like a larger number of trigonometric calculations or indefinite calculation cycles, the on-board classification is cheaper than just transmitting everything.

### Pilot data collection

Collecting and annotating animal-borne data introduces unpredictable challenges with respect to the data output, which would limit our ability to interpret the impact of our methodology. Therefore, we conducted a pilot data collection using human volunteers as a controlled proxy system, instead. The purpose of the experiment is, given a dataset with well distinguishable behavioural states, to be able to estimate and explain potential energy cost reductions when using on-board machine learning. We wanted to be able to read basic movement types from a labelled data set, train a classifier, see how the system behaves, and deploy it on the WildFi tag, thus teaching it to recognise one specified type of movement. For this, we chose 5 behaviours: lying, sitting, standing, walking, and running.

We had 3 colleagues whose movements we tracked with the WildFi tags. We will further refer to the three tags as EA60, EBF8, and ED3C, which are the last four digits of their respective IDs. Each of the devices was encased inside a tight-fit casing, which in turn was attached centrally to a baseball cap’s brim. To record the data, all 3 participants wore these baseball caps. Thus, to refer to participants 1,2, or 3, we can also refer to the respective tag IDs (Table 2). The participants had to fulfil tasks which encompassed the 5 different movement types. The tasks varied in duration but were controlled to last approximately full minute intervals. The entire recording has a duration of about 31 minutes. Between every two tasks, a short transition phase took place, which has varying lengths due to individual differences in movement habits, like how long it takes an individual to stand up, for example. The procedure was video recorded solely to assist labelling to reduce outliers for the training data. It should be noted that for this study, which serves as a proof-of-concept demonstration rather than a statistically robust population analysis, n = 3 is enough. For preparing field studies practitioners require a larger sample of recorded data.

**Table 2:** Data overview.

Tag ID	EA60	EBF8	ED3C
Participant	1	2	3
# Data points	2350	2320	2340
≈ Data size	1410kB	1392kB	1404kB
% Lying	21.97	21.3	21.98
% Sitting	11.58	11.82	11.93
% Standing	17.62	17.9	12.44
% Walking	41.51	41.57	46.47
% Running	7.32	7.42	7.18

This table shows an overview of how the labels in the datasets of the different sensors are distributed. It shows the overall data points per sensor, the data size of the data set as it would have been transmitted, and the percentage of data points per behaviour.

The produced data sets were then decoded and post-processed to remove measurements that did not lie within the period of the experiment, as well as the transition times between two target behaviours. Finally, they were labelled using the video recording.

For the generation of the decision trees, we chose a total of 8 features to train with. All six basic features stemmed from the IMU, specifically the accelerometer and the gyroscope. From the accelerometer, we used the mean per reading of all three axes (AX, AY, AZ), as well as the calculated VeDBA score as a well-established metric. From the gyroscope, we used the variance per reading of all three axes (GX, GY, GZ), as well as the VeDBA calculation function applied on the axes, which we named GVeDBA. The latter is not a common metric, and we were experimenting with it as a sort of energy expenditure equivalent from rotation instead of translation. This has been a purely experimental feature without established biological meaning. We kept it included in our feature space, however, since it produced promising results consistently. Generally, simple statistical features were chosen to minimize computational overhead while establishing baseline performance for this proof-of-concept study.

We generated decision trees for each of the participants, with tree depths of 7 and 14, based on their respective data, and compared the trees with all available features to those with only a subset of features by evaluating their confusion matrices. The behaviour we targeted to optimise was “standing.” Apart from the decision trees and confusion matrices for all trees, we also produced rankings for the different permutations of features based on their performance for recognising the targeted behaviour. Lastly, we estimated potential savings in transmission time based on the outcomes.

## Results

**Fig 4:**
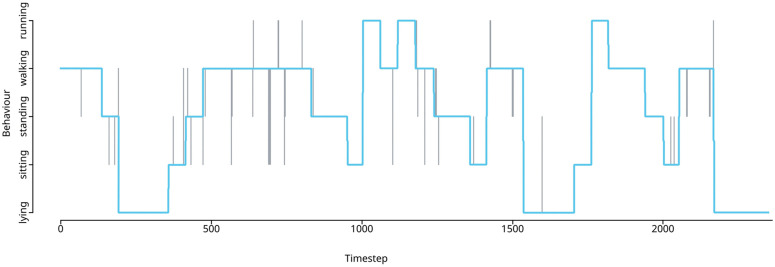
Classifications over timesteps. This plot compares the actual behaviours with the classified behaviours throughout the experimental data. The y-axis indicates the different behaviours. The x-axis shows the timesteps. The blue line shows which behaviour was exhibited during the recording, therefore mapping to the actual labels of the time series data. The grey lines connect the behaviour classified by the decision tree with the actual behaviour at that particular timestep. Therefore, the grey lines indicate wrong classifications.

The datasets we produced, after preprocessing, have 2350 data points for EA60, 2320 for EBF8, and 2240 for ED3C. The overall distribution of the 5 behaviours per individual can be seen in Table 2, with walking being the majority and running the minority class. The approximate data size per individual stems from the number of data points multiplied by the sampling rate of the tri-axis accelerometer and gyroscope, thus, 6·50Hz·2B·#timesteps. Fig 4 illustrates the different phases of the underlying time series, by mapping the respective behaviours (y-axis) to the points in time when they happened (x-axis) with a blue line.

The training of the decision trees produced a total of $2^{n}-1=255$ trees per participant. The F1 scores and accuracies of the best performing feature sub-sets, as well as the full feature sets, can be seen in Table 3 for each individual and for the two tree depths 7 and 14, respectively. For those, we also produced header files which can be used by the WildFi to classify the behaviours on board directly by using the sensor values. The resulting header files have a size of ≈4.9kB on average for a tree depth of 7 and ≈21.5kB on average for a tree depth of 14, which comfortably fits onto the WildFi tag. The full feature set shows a higher F1 score than accuracy consistently over individuals and tree depths, while the feature subsets consistently have higher accuracy than the F1 score. Also, each tree depth 14 version surpasses the tree depth 7 version in terms of F1 and accuracy. Furthermore, for each individual, it holds that a subset of features holds a higher accuracy compared to the full feature set while showing a lower F1 score. Notably, the feature subset for Participant 3 with a tree depth of 7 completely outperformed the full feature set.

**Table 3:** Output results during testing.

Tag ID &	Full Set	Full Set	Subset	Subset
Tree depth	F1	accuracy	F1	accuracy
EA60 TD 7	79.37%	65.90%	66.01%	79.46%
EA60 TD 14	92.34%	85.81%	87.24%	93.16%
EBF8 TD 7	73.33%	58.01%	60.02%	74.93%
EBF8 TD 14	90.04%	81.94%	83.22%	90.80%
ED3C TD 7	62.62%	45.74%	69.56%	82.00%
ED3C TD 14	96.34%	92.96%	93.22%	96.48%

The performance metrics of the different decision trees during the testing phase. Shown are the F1 score and the accuracy of the full feature space and the best-performing feature subset per individual, for tree-depths (TD) 7 and 14, respectively.

Table 4 shows some permutations of EA60 with a tree depth of 14 together with their ranking in terms of quality of classification for the behaviour “standing.” For the full table, as well as all code used for (pre-)processing our project and data therein, please refer to the supplementary material that can be downloaded from our Zenodo repository [56]. The first finding to notice is that we obtain a higher accuracy as well as a higher F1 ratio while not using all available features. In fact, the decision tree that uses all features ranked in the twentieth place. Also noteworthy is that the gyroscope is included with at least one axis in the first 200 of 256 configurations. Rank 38 is the first permutation that only requires 3 features and still offers a satisfying accuracy of 90.47%. A last observation is that rank 3 is less than 1% less accurate than rank 1, but requires a whole axis less to achieve this, which is 20% less relevant data to record and or transmit.

**Table 4:** A shortened overview of feature permutation performances.

n-^th^ best	Feature Permutation	F1 in %	Accuracy in %
1	GX;GY;GZ;AX;AZ;	87.24	93.16
2	GX;GY;GZ;AX;AZ;	87.04	93.04
	GVeDBA;		
3	GX;GY;GZ;AZ;	85.95	92.41
4	GY;GZ;AX;AY;	85.31	92.04
5	GY;GZ;AY;VeDBA;	85.02	91.87
...	...	...	...
20	GX;GY;GZ;AX;AY;	83.81	91.16
	AZ;VeDBA;GVeDBA;		
...	...	...	...
38	GX;GZ;AX;	82.65	90.47
...	...	...	...

This table shows a selection of feature permutations for EA60 with a tree depth of 14, with their respective F1 and accuracy scores. The “n-th best” column signifies the rank of the respective feature permutation regarding its quality measures for classifying a priorly specified behaviour. In this case, this behaviour is “standing.” The full table is included in the supplementary materials.

**Fig 5:**
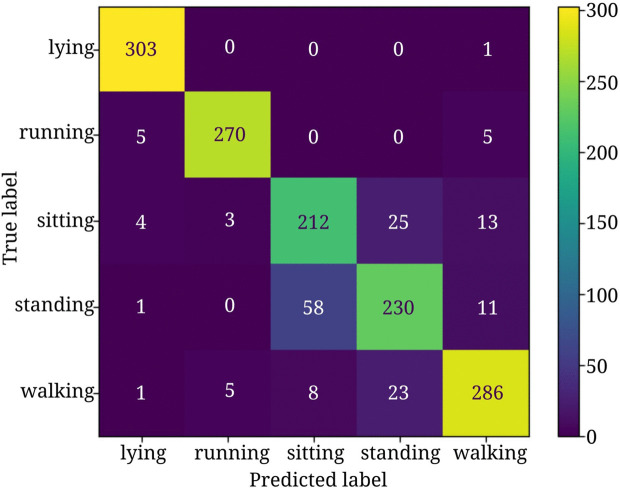
Confusion matrix with tree depth 7 for EA60. This image shows the confusion matrix for the decision tree, optimised for the behaviour standing, with a tree depth of 7, belonging to Participant 1. The decision tree has an increased number of misclassifications between sitting, standing, and walking. The y-axis shows the actual behaviours, and the x-axis shows what the classifier has predicted.

To illustrate the actual performance of the decision tree, we exemplarily illustrate the classification of the EA60 tree using a feature subset and a tree depth of 14 in Fig 4. There, the grey lines connect the classified behaviour and the actual behaviour at that time step, such that it becomes visible when and how often the classifier got its classification wrong. It quickly becomes visible that the classifier had the most problems with seeing the difference between sitting, standing, and walking. The respective confusion matrix in Fig 5 shows that this problem becomes even more evident for a tree depth of 7, especially for the differentiation between standing and walking. As a means of cross validation, we applied the classifier with a tree depth of 7 and 14, respectively, created for EA60 on ED3C. Those result in an accuracy of 27.13% for tree depth 14 and 25.44% for a tree depth of 7.

Since there are only a few false negatives for “standing,” we now assume the classifier is feasible to detect that behaviour, and we can conduct some example calculations for energy saving for the WildFi tag. For conditional data transmission, taking Table 2 as a basis and considering “standing” to occur with an estimated frequency of 17.62% for EA60, the WildFi tag would now be able to transmit information with an estimated frequency of 17.62%. Therefore, only 2350·0.1762≈414 data points of the time series would be appointed for saving and transmission, reducing the overall message length from 1410kB to 248.4kB and the consumed energy from $\frac{3.75V\cdot 0.108A\cdot 1410kB}{2.3\cdot 10^{5}B/s}=0.405W\cdot 6.13s=2.48265J$ to $\frac{3.75V\cdot 0.108A\cdot 248.4kB}{2.3\cdot 10^{5}B/s}=0.405W\cdot 1.08s=0.4374J$.

As selected data transmission is versatile, for our calculation, we will assume that we only transmit the information that leads to the classification. In that case, and concerning Table 4, the solution with the highest accuracy would exclude one axis of the accelerometer readings. Since the initial amount of input features suggested six IMU features, which record with a rate of 600B/s, the reduction of information to transmit of the IMU lies at $\frac{1}{6}$. The approximate data size for EA60 in the given scenario reduces to 1175kB, accordingly, with a reduction of power consumption to $\frac{3.75V\cdot 0.108A\cdot 1175kB}{2.3\cdot 10^{5}B/s}=0.405W\cdot 5.11s\approx 2.06J$.

With conditional and selective, we reach a total reduction to $0.1762\cdot \frac{500}{600}\approx 0.1468=14.68\%$. Thus, we only transmit 14.68% of the amount of data compared to transmitting all of the raw data from the IMU, which equals a consumption of ≈0.36J. The second, more extreme, example is to only send a 2B sized signal (using the unsigned short data type), the timestamps for when a target behaviour is detected. In that case, the total transmitted data is reduced to 2 times the frequency of a behaviour times the number of data points. For our example, that would be 2·0.1762·2350≈828B such that the final transmission costs are reduced by 99.9903%, which we consider to be an upper bound.

## Discussion

The main goal of this study is to investigate how to utilise machine learning for reduced energy consumption in the context of data transmission on animal-borne devices that use WiFi as a transmission protocol. To approach this problem, we simplified the energy consumption of data transmission. We made it directly relate to the transmission time and omitted energy consumption overhead caused by different aspects, such as the time required for the transceivers to connect. To shorten transmission time, we in turn aimed for a reduction of the overall amount of data transmitted. We argued that there are several ways to achieve this, like data compression, encoding, or filtering. In this study, we concentrated on the filtering aspect and partly utilised encoding, but without an emphasis on it. Consequently, in future work, more emphasis on data compression and encoding is required. Filtering, as we argued, can happen in the form of either filtering out whole data points, parts of each data point, or a combination of both. We named the two forms of filtering conditional and selective data transmission. For the former, we must decide under which circumstances to transmit. For the latter, we determine what data exactly to transmit. An important limitation here is the fact that this filter only applies to what can be sent, once the transmission overhead is not neglected, but not when something is being sent. If we cut the data into many small pieces and send them via WiFi, with the need to open and close a transmission channel for every message, the energy preservation is either partly, if not fully, lost. Each transmission adds additional energy costs of $E_{c}\approx 1.2J$ which may be acceptable to some extent, but adds up quickly over time in relation to the energy costs of the transmission process $E_{tx}$. This fact only underlines the desire to minimise the amount of transmissions, thus supporting the filtering approach even further. Yet, a limitation of behaviour-based filtering is the potential loss of rare, but ecologically relevant behaviours. If target behaviours occur infrequently, the filtering may exclude events that would have been of interest, thus necessitating active consideration of this possibility during the design phase.

To make decisions about what to filter, we used machine learning in the form of pattern recognition. Specifically, certain states of an individual, namely their behaviours, could aid in making a distinction between what to send, or store, and what not. Since the identification and classification of animal behaviours from time series data has gained popularity in the past, also due to the increased availability of well-performing bio-loggers, we focused on that.

To achieve classifications, as we wanted to study the potential behind machine learning for energy preservation, we decided to use decision trees. Their structure, which is very simple compared to more elaborate models like deep neural networks, or more sophisticated TinyML methods, such as random forests, allows us to control the number of computations per classification directly and to interpret what exactly impacted their quality. More powerful models would very likely yield better classifications. They could even employ adaptive strategies, such as the work of Jin et al. [57]. However, they add layers of complexity in explainability, which is an essential component of this work. Some, such as deep neural networks, may fall short in explainability entirely, rendering approximations of potential gains in terms of battery lifetime a pure matter of measurements on the device. Another benefit is that decision trees, due to the way they work, can be translated into simple pieces of code, which are small enough to be deployed on and operated by devices that are lightweight by design. We made use of this fact and derived code files for the classification models we obtained that we were able to deploy on the bio-logger, which we used – the WildFi tag [20]. Thanks to the high computational capabilities of these state-of-the-art bio-loggers, we can therefore do classification on the bio-logger, which in itself presents a significant benefit to the field of bio-logging. However, decision trees are not nearly as powerful as other machine learning models concerning recognising behaviours, so further investigation on how more complex classifiers could be used on devices like the WildFi tag is required. Also, we consider classifications as static for our considerations. However, animal behaviour changes due to various factors, such as environmental factors or season. So, to utilise what we discuss in this work, practitioners should consider works from IoT research, such as from Luo et al., who discuss context aware adaptations [58].

To produce pattern recognition models, from the computer science perspective, we were guided by the activity recognition chain proposed by Bulling et al. [50], such that our general approach has a sound foundation. Due to our goal to preserve energy, we outsourced as many steps as possible from the loggers to a PC. Thus, although possible, we did not train our decision trees on the WildFi. Furthermore, we incorporated parts of the work of Williams et al. [51] about interdisciplinary work on animal behaviour to account for the complexity of animal behaviour-related topics. Through the integration of their findings about which sensor types are relevant for which kind of question, the experiment we conducted gained further insights and validity. As an easy proof of concept, we conducted a small experiment by recording human data, applying our workflow to it, analysing the results, and inferring the practicality of it, as well as gaining some numbers to do example calculations. The data we recorded consists of roughly 30 minutes of data points per participant and mirrors five different and distinct behaviours. We chose the behaviours such that they would all be patterns in body posture, hence requiring intrinsic sensor types. Furthermore, they were all mutually exclusive for the most part, thus simplifying the data set. As a consequence, our experiment has limitations in the sense that we do not fully depict a realistic scenario of actual deployments of animals, that face additional challenges. Different behaviours might overlap. A cat might simultaneously walk and chew, for instance. Such overlap of different behaviours may have benefits in terms of filtering, as ecologically rare behaviour may be included in the collected data. Vice versa, however, such rare behaviour, like predator encounters, may be discarded, even though of interest, if those are not part of the data that the classifier deemed to be relevant. The lossiness of data is part of the design, but must be considered carefully to avoid undesired discarding behaviour of the logger. Another limitation is posed by environmental factors. Variations in temperatures, humidity, precipitation, or attachment stability influence the performance of such systems. While animals groom, they may change the positioning of the sensors. Rain can negatively influence the transmission rate, changing humidity or temperatures may influence the behaviour of the tracked animal. Some of those cases can be tackled by the aforementioned adaptive strategies in further developments that merge our considerations with works that prioritise classification performance.

However, since we only want to investigate the potential of machine learning in this study, we deliberately designed a simpler use case, to capture the raw potential. This is also the reason why we only trained on human data. It is easier to control and interpret, which gives us enough opportunity to look into how the relative energy expenditure behaves.

To gain an even deeper insight, we not only look into general decision trees, but we also trained a number of them with different depths and all possible feature combinations to see which sensor may have more or less impact on successfully identifying behaviours. Our experimental results suggest that the decision trees with a maximum tree depth of 14 are capable of classifying with high accuracy. This was to be expected since 14 is likely an overfit, as trying to apply the same tree trained on another individual results in poor accuracy. Therefore, a tree depth of 14 mirrors the specific behaviour of one individual, instead of being generalisable. However, tree depths of 7 still maintain relatively high accuracy with 82% for ED3C, for example. These outcomes can likely be further improved with different features or more data than only half an hour of data points, or by employing more sophisticated sampling methods than oversampling. Generally, our classification results would benefit from follow-up studies, where the focus is on using data from more individuals to help overcome the inability to use a classifier inter-individually. With data from a larger number of individuals with the same quality, the individuals’ respective movement idiosyncrasies, like habit-, gender-, or age-dependent nuances, would not impact the classifier as much. We might have been able to use the resulting classifier on individuals that weren’t part of the recording with higher accuracy than the deficient accuracy we had between EA60 and ED3C. An interesting approach to further improve the use of decision trees would be to use transfer learning between individuals during runtime for animals where this is feasible (i.e., not hard to reach). Decision trees could be deployed while only having a feasible foundation in terms of precision and be trained using the individuals’ ideosynchracies [59]. Generally, not only do different individuals have their own movement habits. Most of the different species likely have vastly different patterns for the same behaviour, necessitating species-specific experimentation, utilising, for instance, the general approach we present. For example, our head-mounted sensor may not reflect typical bio-logger deployment. Animal bio-loggers are often attached to the back or limbs, depending on the species.

Another interesting finding has to do with the feature space. Specifically, the gyroscope proves to be important for behavioural recognition. It appears in all of the best-performing configurations of our feature space for “standing.” An explanation could be that while an individual is idly standing, their head will move around more than during a running action. And since the bio-logger was effectively attached to the head, this would result in more changes in rotation and less in acceleration, which the classifier would use to recognise the behaviour. Comparing the findings of Pozzo et al. [60] and Hirasaki and Kumakura [61], who investigate head movements of Humans and Macaque monkeys, respectively, it becomes evident that different species show different angles of head rotation during movement. This fact reinforces that data that gyroscopes can identify well, namely angular change, is at least relevant to distinguish between species. Generally, some systematic and context-dependent head movement is likely linked to certain behaviours, which the gyroscope captures in a different granularity than the accelerometer. This appears to help in distinction, thus rendering the gyroscope important for classification purposes. Apart from the exact quality metrics, it becomes clear that classifiers that work for individuals on bio-loggers appear to be possible with only about 30 minutes’ worth of data. How long a time series has to be in the end depends on the nature of the targeted behaviour, external influences, and further factors, however.

Under the assumption of a classifier that could predict “standing” reliably, we did some example calculations for possible energy savings based on a hypothetical scenario, where the stored and transmitted data is limited to the data points required to detect a given target behaviour. We predicted that the transmission time for sending only occurrences of the behaviour “standing” together with 5 features would be reduced to approximately 14.68%. The actual impact on the overall runtime of the WildFi tag depends on the setup, however. Assuming Table 2 from the WildFi’s original publication [20], the increase in energy consumption ranges from as little as 0.03% to 58%. A similar reduction of the 58% would leave us with only 8.51% extra cost through transmission, thereby increasing the runtime of the device from 94 to about 137 days, giving researchers over a month of extra time to collect relevant data. What is more, the higher the impact of the transmission, the higher the gain of smart choices in what to transmit. A big limitation here is that there are a lot of circumstances that influence the results. For one, classifiers do not offer 100 per cent solutions. Their reliability depends on the data, the actual behaviour of the animal they operate on, the way the classifier model was created, and more. Also, the software and hardware setup of a bio-logger determines the impact such machine-learning solutions can have and how extensive they can be. For example, we discussed the approximate energy expenditure of a single operation. These costs highly influence how extensive operations on a bio-logger can be before they exceed the costs for transmitting the raw information. Another fact to consider about the classification costs is that our cost estimate for a single simple classification could realistically lie at roughly $3.753\cdot 10^{-6}J$ and a single transmission establishment at 1.2*J*. Consequently, we quickly see the limitation of selective transmissions. In field applications, such bio-loggers use transmission bursts, in which they only transmit a full data burst upon reaching predefined criteria. For our approach, this only means that, instead of transmitting upon identifying a certain behaviour, the storing is controlled and, by this, the transmission becomes cheaper by sending less or less frequently.

As a last remark for future work, more than only on-board classification should be assessed to further press the energy consumption reduction idea in the form of a more generalised inspection that incorporates network-level considerations. Strategies that reduce the overhead of transmissions intelligently could be looked further into [62]. Also, the work by Liu et al. presents a new design paradigm for duty-cycling communication that could be highly relevant [63]. Their approach demonstrates how duty-cycling can be optimized to reduce energy consumption without sacrificing network performance. When several individuals with sensors attached to them that are close to each other, those sensors may interfere with each other, ultimately requiring more energy to provide dependable results. This may be addressed by dedicated approaches akin to the work of Cao et al [64], who propose elaborate models to approach this issue.

Despite the various factors of influence and even though omitting certain sets of data points can also be achieved through other means than machine learning, the possibility of using classifiers as an onboard solution offers more options since elaborate contextual decisions can be achieved. For example, bio-loggers could react in situ and send warnings if an animal exhibits unusual behaviour, enabling responsible persons or scientists to react in a timely manner. Also, such classification approaches could be used to govern the very limited transmission capabilities of devices that use SigFox as a transmission protocol. By that, the high transmission range could be utilised much more efficiently, as selective transmission yields even greater relative benefits for systems with more restrictions, such as SigFox. With these insights, we combined the works of several researchers, such as Korpela et al., Bulling et al., and Williams et al. We showed that given a capable bio-logger with WiFi technology, machine learning is not only possible on such a device, but is also feasible for significant current consumption reduction through smart decision-making on when to transmit which information.

## Conclusion

In this study, we explored the potential of using well-established and simple machine learning, specifically decision trees, to optimize energy consumption in WiFi-enabled bio-logging devices that suffer from increased energy demand compared to those that don’t use that transmission protocol. We do so by using decision trees as filters for the data we want to transmit. We did this exemplarily through the use of on-board classification on the WildFi bio-logger. By focusing on the identification of behaviours through machine learning, we utilized features derived from accelerometer and gyroscope data to train decision trees of varying depths and combinations of sensor values and their encodings. We conducted an experiment to gain data that held respective sensor values and produced the varying decision trees. Furthermore, we investigated the impact of the different sensors and their encodings on some decision trees’ accuracy metrics. Our experimental results indicate that decision trees can effectively classify behaviours with high accuracy while maintaining reasonably high performance on board. A key finding is the importance of gyroscope data, which consistently appeared in the top-performing feature combinations, particularly for distinguishing between behaviours such as standing and walking. This suggests that rotational data, as captured by the gyroscope, plays a critical role in behaviour recognition, which makes sense from a data-analysis perspective. In the context of animal-borne devices, the implications pose a direct asset, however, as using the gyroscope can actually help to produce more meaningful data while simultaneously reducing the energy expenditure. Our results suggest that this happens once the data to transmit is carefully selected under utilization of the output of decision trees that, in turn, use, among others, said gyroscope data. By reducing the amount of data transmitted to only what is necessary for behaviour classification, we predicted significant theoretical reductions in energy consumption—ranging from 14.68% in our artificial combined filtering scenario to 99.9903% in extreme theoretical cases where only timestamps are transmitted. This energy consumption reduction has profound implications for the operational longevity of WiFi-capable bio-loggers, potentially extending their runtime from 94 to 137 days, as is the case for one example configuration of the WildFi tag, which is widely used by the Max Planck Institute of Animal Behavior, for instance. Thus, our findings bring together the feasibility and potential benefits of integrating machine learning models into bio-loggers for real-time, on-board data processing and energy-efficient operation for the WildFi tag. Our proof-of-concept indicates that on-board classification on bio-loggers and the use of it as a data filter shows promise, that it is feasible within reasonable limits, and that it can significantly increase the runtime of a device.

In conclusion, this study contributes to the growing body of research on the intersection of animal behaviour monitoring and machine learning, offering insights into how even simple machine-learning approaches can be utilised to make bio-logging more energy-efficient, thereby enabling longer-term and more detailed wildlife studies.
