# Supplementary material for: Resource-efficient data transmission for WiFi-capable bio-loggers based on machine learning
Source: PLoS One. 2026 Jul 24;21(7):e0354146. doi: 10.1371/journal.pone.0354146 (PMC13399348; doi:10.1371/journal.pone.0354146)
Supplement: Attachment — Submitted filename: renamed_fdb09.docx [file pone.0354146.s001.docx]

**🔴 TITLE & SCOPE**

1. The study is validated using controlled human data. How do the authors justify generalizing conclusions to animal-borne bio-loggers?
2. Why does the title imply broad applicability to WiFi-enabled bio-loggers when experiments were conducted only on the WildFi tag?
3. Does the title accurately reflect that the study evaluates shallow decision-tree–based ML rather than a broader AI framework?

**🔴 ABSTRACT**

4. The abstract claims energy costs are reduced to “a tenth.” Under what behavioral frequency assumptions was this calculated?
5. How robust are the reported >80% accuracies given the small sample size (n = 3)?
6. Why are no confidence intervals or uncertainty estimates reported for performance metrics?
7. Is the statement that gyroscopes are “less commonly utilized” supported by literature evidence?

**🔴 INTRODUCTION**

8. What is the precise novelty compared to prior onboard ML work (e.g., Korpela et al.)?
9. How does this approach differ from embedded TinyML systems such as those implemented using TensorFlow Lite?
10. Why was WiFi selected as the focal communication protocol instead of lower-power alternatives?
11. Have the authors quantitatively compared their method against compression-only or adaptive duty-cycling strategies?

**🔴 MATERIALS & METHODS**

**Study Design**

12. Why were only three human participants included?
13. Was cross-validation (e.g., leave-one-subject-out) performed to test generalization?
14. Were training and testing datasets strictly separated to prevent data leakage?
15. How was overfitting assessed, particularly for tree depth 14?

**Feature Engineering**

16. Why were only simple statistical features (mean and variance) used?
17. Were time-domain and frequency-domain features evaluated?
18. Is the proposed GVeDBA metric physiologically validated?

**Modeling**

19. Why were only decision trees tested?
20. How does performance compare with lightweight ensemble models (e.g., random forest)?
21. How was hyperparameter tuning conducted?

**Onboard Deployment**

22. Was real onboard power consumption measured experimentally?
23. What is the memory footprint of the deployed model on the MCU?
24. Was classification latency measured under real deployment conditions?

**🔴 ENERGY CALCULATION**

25. Why does the energy model ignore WiFi connection and handshake overhead?
26. Are the reported 99.9903% energy savings realistic under field transmission conditions?
27. Why is energy reported in mA rather than Joules, and were battery discharge characteristics considered?

**🔴 RESULTS**

28. Why is inter-individual generalization as low as 27%, and what does this imply for deployment?
29. Were statistical significance tests performed between models?
30. Why are uncertainty measures (confidence intervals) absent?

**🔴 GENERALIZATION TO ANIMALS**

31. Human head-mounted motion differs substantially from animal-mounted tags. How transferable are these results?
32. How would species-specific gait variability affect classifier performance?
33. Have any pilot deployments been conducted on animals to validate applicability?

**🔴 DISCUSSION**

34. Why are extreme theoretical energy savings emphasized rather than realistic deployment scenarios?
35. Does behavior-based filtering risk discarding rare but ecologically important behaviors?

**🔴 LIMITATIONS**

36. The manuscript is framed as proof-of-concept, yet conclusions are strongly worded. Is the framing proportional to the evidence?

**🔴 DATA & REPRODUCIBILITY**

37. Is the full firmware source code publicly available?
38. Can the decision trees be exactly regenerated from the shared dataset?

**🔴 ETHICS**

39. Since human subjects were recorded, was institutional ethical approval obtained?
40. Was informed consent documented?

**🔴 OVERALL CRITICAL QUESTION**

41. Does this study demonstrate a generalizable, field-validated system — or a controlled laboratory proof-of-concept limited to one device and three human subjects?
