# Supplementary material for: Resource-efficient data transmission for WiFi-capable bio-loggers based on machine learning
Source: PLoS One. 2026 Jul 24;21(7):e0354146. doi: 10.1371/journal.pone.0354146 (PMC13399348; doi:10.1371/journal.pone.0354146)
Supplement: Attachment — Submitted filename: Response to Reviewers.docx [file pone.0354146.s003.docx]

Dear esteemed Colleagues,

Thank you very much for the opportunity to revise and resubmit our manuscript to PLOS ONE. We appreciate the constructive feedback provided by the editor and reviewers. Their comments helped us improve the clarity, rigor, and framing of our work substantially.

With respect to the comments, we would like to highlight the following changes and clarifications.

Editing issues
**1. Please ensure that your manuscript meets PLOS ONE's style requirements, including those for file naming.** 
- We adapted the manuscript to match the PLOS ONE style templates to the best of our knowledge. This includes, but is not limited to, adapting the figure captions, reformatting the figures, and shortening the abstract to comply with the 300-word restriction. Please let us know if any additional formatting adjustments are required, and we will address them accordingly.
**2. Please note that PLOS One has specific guidelines on code sharing for submissions in which author-generated code underpins the findings in the manuscript. In these cases, we expect all author-generated code to be made available without restrictions upon publication of the work.**
- Regarding code sharing, we improved our Zenodo repository to facilitate reproducibility. The updated DOI has already been incorporated into the manuscript. Specifically, we cleaned up legacy code components and added a README file to improve usability and reproducibility.
**3. We note that you have provided funding information that is not currently declared in your Funding Statement. However, funding information should not appear in the Acknowledgments section or other areas of your manuscript. We will only publish funding information present in the Funding Statement section of the online submission form.** 
- The detailed funding information has been removed from the Acknowledgements section, in accordance with the journal guidelines, and we understand that it will instead be included in the appropriate funding section during production.
**4. We note that the grant information you provided in the ‘Funding Information’ and ‘Financial Disclosure’ sections do not match.** 
- Thank you very much for pointing this out. The correct funding information is 1: DFG, project ID 251654672--TRR 161; 2: Academy for the Protection of Zoo Animals and Wildlife e.V and the Gordon and Betty Moore Foundation grant number GBMF10539. We will ensure that this information is correctly reflected during resubmission.

For the Acknowledgments section, the following funding statement is the correct one:
“We acknowledge funding by DFG, under Germany’s Excellence Strategy – EXC 2117 – 422037984, and DFG project ID 251654672 – TRR 161. Furthermore, we acknowledge funding support from the Academy for the Protection of Zoo Animals and Wildlife e.V., and the Gordon and Betty Moore Foundation through grant GBMF10539.”
**5. Please state what role the funders took in the study.  If the funders had no role, please state: "The funders had no role in study design, data collection and analysis, decision to publish, or preparation of the manuscript."** 
- Thank you for identifying this issue. We added the proposed statement: "The funders had no role in study design, data collection and analysis, decision to publish, or preparation of the manuscript."
**6. We require you to either (1) present written permission from the copyright holder to publish these figures specifically under the CC BY 4.0 license, or (2) remove the figures from your submission**
- Permission for Figure 1 has been granted by the copyright holder, and we uploaded the corresponding permission form. We also added the required note to the figure caption.


Reviewer #1

**1. The manuscript focuses heavily on device-level energy savings through data filtering but does not adequately address network-level energy optimization strategies that could complement the proposed approach. While the authors mention IoT strategies briefly, the discussion remains primarily at the individual logger level. Consider incorporating findings from [Xu et al. (2022): Energy Consumption Minimization for Data Collection From Wirelessly-Powered IoT Sensors: Session-Specific Optimal Design With DRL. IEEE Sensors Journal, 22(20), 19886-19896. doi: 10.1109/JSEN.2022.3205017] which presents energy consumption minimization techniques for data collection from wirelessly-powered IoT sensors. Their session-specific optimal design using deep reinforcement learning could provide valuable context for optimizing when and how bio-loggers transmit data, especially in multi-device deployment scenarios common in animal behavior studies. Add this discussion in Section 2.2 (General Concepts) or the Discussion section when addressing transmission overhead considerations.**
- We thank the reviewer for this valuable suggestion. While network-level optimization is beyond the scope of our proof-of-concept study, which primarily focuses on device-level energy consumption minimization, we agree that adaptive transmission strategies are highly relevant for broader deployment scenarios. We therefore added a dedicated paragraph in the Discussion section addressing future improvements in this direction.

**2. The manuscript acknowledges WiFi's higher energy cost compared to lightweight protocols but does not explore potential improvements at the communication protocol level that could further reduce energy consumption while maintaining WiFi's advantages. At Section 2.2 (General Concepts) or Section 5 (Discussion) when addressing transmission overhead and connection time considerations, add this statement: The work by [Liu et al. (2022): Concurrent Low-Power Listening: A New Design Paradigm for Duty-Cycling Communication. ACM Trans. Sen. Netw., 19(1). doi: 10.1145/3517013] on Concurrent Low-Power Listening presents a new design paradigm for duty-cycling communication that could be highly relevant. Their approach demonstrates how duty-cycling can be optimized to reduce energy consumption without sacrificing network performance. This could inform future iterations of the WildFi tag's communication strategy.**
- We thank the reviewer for this helpful input. Bio-loggers are frequently deployed on multiple individuals in close proximity. Since WiFi-capable loggers already commonly rely on gateways to reduce WiFi connection costs, considerations regarding interference avoidance are highly relevant. We therefore incorporated this aspect into the Discussion section.

**3. The authors note that classifiers trained on one individual perform poorly on others (e.g., EA60 classifier on ED3C yielded only 27.13% accuracy). This limitation is acknowledged but not adequately addressed in terms of potential solutions. Recent advances in transfer learning could offer pathways to address this limitation. At Section 5 (Discussion) when addressing inter-individual classifier performance limitations. consider citing [Liu et al. (2025): Establishing a Digital Twin Diagnostic Model Based on Cross-Device Transfer Learning. IEEE Transactions on Instrumentation and Measurement, 74, 1-10. doi: 10.1109/TIM.2025.3562973] which presents a digital twin diagnostic model based on cross-device transfer learning. Their approach demonstrates how models can be adapted across different devices or individuals, potentially enabling more generalizable behavior classifiers. Additionally, [Yan et al. (2025): A Generalizable Load Recognition Method in NILM Based on Transferable Random Forest. IEEE Transactions on Instrumentation and Measurement, 74. doi: 10.1109/TIM.2025.3570355] on transferable random forests for load recognition could provide insights specific to tree-based models like those used in this study.**
- We appreciate the reviewer’s suggestion regarding transfer learning approaches for improving inter-individual performance. We added a paragraph in the Discussion section addressing transfer learning using decision trees. While the suggested publications raise important points, approaches such as random forests introduce additional computational and energy complexity for onboard deployment. A detailed exploration of these methods would extend beyond the scope of the present proof-of-concept study.

**4. The manuscript treats behavior classification as a static problem, but animal behaviors often exhibit temporal patterns and may change over time due to environmental factors, habituation, or physiological states. At Section 3.3 (Feature Calculation) or Section 5 (Discussion) when addressing the complexity of animal behavior patterns, consider adding: Two recent papers could strengthen this aspect. First, [Wu et al. (2025): Tidal-Like Concept Drift in RIS-Covered Buildings: When Programmable Wireless Environments Meet Human Behaviors. IEEE Wireless Communications, 1-8. doi: 10.1109/MWC.2025.3600792] discusses concept drift in programmable wireless environments when human behaviors change - a relevant consideration for long-term animal behavior studies where behavioral patterns may shift. Second, [Luo et al. (2025): A2Tformer: Addressing Temporal Bias and Non-Stationarity in Transformer-Based IoT Time Series Classification. IEEE Internet of Things Journal, 1. doi: 10.1109/JIOT.2025.3595765] addresses temporal bias and non-stationarity in transformer-based IoT time series classification, offering methodological insights that could be adapted for bio-logging applications.**
- We thank the reviewer for this valuable input. We agree that concept drift can be highly relevant in this context, particularly for long-term deployments such as studies on migratory animals. While the first proposed work focuses primarily on indoor applications and therefore aligns less directly with our use case, the work by Luo et al. fits well within our discussion and has been incorporated accordingly.

**5. The manuscript focuses on single-device energy savings but does not address scenarios where multiple bio-loggers operate in proximity (e.g., studying social animals in groups). In such cases, network-level coordination could yield additional energy savings. The work by [Cao et al. (2021): RFID Reader Anticollision Based on Distributed Parallel Particle Swarm Optimization. IEEE internet of things journal, 8(5), 3099-3107. doi: 10.1109/JIOT.2020.3033473] on RFID reader anticollision using distributed parallel particle swarm optimization demonstrates how multi-device interference can be managed. While their context differs, the principles of coordinating multiple transmitting devices could inform future extensions of this work for group-deployment scenarios.**
- We agree that this work is relevant and have added it to the Discussion section.

**6. The manuscript mentions on-board classification but does not discuss the broader ecosystem of edge computing that could support more sophisticated processing while maintaining energy efficiency. At Section 2.2 (General Concepts) when discussing transmission protocols, or Section 5 (Discussion) as a future direction. Consider adding [ Xu et al. (2025): MPAEE: A Multipath Adaptive Energy-Efficient Routing Scheme for Low Earth Orbit-Based Industrial Internet of Things. IEEE Internet of Things Journal, 12(17), 34793-34805. doi: 10.1109/JIOT.2025.3581314] presents a multipath adaptive energy-efficient routing scheme for low Earth orbit-based industrial IoT. While specifically focused on satellite communication, their approach to adaptive, energy-aware routing could inspire similar strategies for bio-logging data transmission, particularly for loggers that may need to route data through intermediate nodes.**
- We agree that this work is relevant in the context of broader energy-preservation ecosystems. However, it is less directly applicable to WildFi bio-loggers specifically, since WiFi-capable bio-loggers typically rely on gateways to avoid routing overhead, which itself can be energetically expensive. We added a short elaboration on this point in the “General Concepts” section as part of our extended discussion of overhead considerations.

**7. The authors demonstrate the effectiveness of decision trees but do not compare their approach with other machine learning methods that might offer better trade-offs for specific behaviors or sensor configurations. When addressing limitations of decision trees compared to more complex models, consider citing [Jin et al. (2022): A fixed-time convergent and noise-tolerant zeroing neural network for online solution of time-varying matrix inversion. Applied Soft Computing, 130, 109691. doi:** [**https://doi.org/10.1016/j.asoc.2022.109691**](https://doi.org/10.1016/j.asoc.2022.109691)**] which presents a fixed-time convergent zeroing neural network for online solutions. While their application differs, their approach to time-varying system modeling could inspire more sophisticated on-board processing techniques that adapt to changing animal behaviors over time. This could complement the decision tree approach presented here.**
- We thank the reviewer for this suggestion. We already discussed alternative approaches briefly in the Discussion section; however, we have now expanded this part and incorporated the proposed work accordingly.

**8. The energy calculations in Section 2.2 are helpful but the assumptions could be stated more explicitly. The authors assume invariant transmission rate and neglect overheads, which is reasonable for initial estimates, but the limitations of these assumptions should be discussed more prominently.**
- We thank the reviewer for this important remark. We addressed this point in greater detail in the "General Concepts" section, where we discuss potential distortions, possible transmission costs, and overhead assumptions more explicitly.

**9. The finding about gyroscope importance (appearing in top-performing feature combinations) is valuable. The authors provide a plausible explanation (head movement during standing vs. running). This could be expanded with more biomechanical reasoning, possibly citing literature on head stabilization during locomotion in different species.**
- We thank the reviewer for this input and provide two works on head stabilization in context - humans and macaques - to show that even between species that are morphologically close to each other significant differences that can be captured with gyroscopes can be observed. Please find the corresponding passage in the Discussion.

**10. The controlled human experiment provides a useful proof-of-concept, but the manuscript would benefit from a more detailed discussion of challenges specific to animal deployment: weather effects on sensors, attachment stability over time, and behavioral variability within and across species.**
- We are grateful for the reviewer’s comment and agree that a more detailed discussion of deployment-related challenges strengthens the manuscript’s perspective on real-world applicability.


Reviewer #2)
We thank Reviewer #2 for their extensive input on our manuscript. We would like to emphasize again the proof-of-concept nature of the present study. The controlled human dataset (n = 3) was not intended to establish a highly generalizable behavioral classifier, but rather to provide a controlled and interpretable dataset that allows us to investigate the conceptual relationship between onboard classification and energy preservation.
We fully agree that the statistical robustness of the presented results is limited by the small sample size, and we have revised the manuscript to emphasize this point more clearly throughout.
Our primary aim was not classifier optimization, but rather the investigation of energy-saving potentials. The extensive discussion of classifier structure and decision trees was included to provide sufficient methodological context for interpreting the energy-saving considerations under practical deployment assumptions.
We also agree that transmission overheads should not be omitted entirely, even in a proof-of-concept study. We therefore expanded the discussion of overheads substantially throughout the manuscript.
Similarly, we now state more clearly that the reported energy reductions represent theoretical rather than practical savings estimates. We additionally clarified the novelty of our contribution, expanded the discussion regarding potentially missed behaviors due to filtering, and added clarification regarding ethical approval.


**1. The study is validated using controlled human data. How do the authors justify generalizing conclusions to animal-borne bio-loggers?**
- The study serves as a proof-of-concept investigation into the energy expenditure of WiFi-enabled bio-loggers. The broader applicability arises from the fact that energy preservation is particularly important in field deployments, where additional environmental influences such as rain or temperature may further increase energy costs. We therefore provide a theoretical and methodological foundation for future field studies, while explicitly acknowledging that species-specific experiments will be necessary in future work.
**2. Why does the title imply broad applicability to WiFi-enabled bio-loggers when experiments were conducted only on the WildFi tag?**
- The broader title reflects that we propose a general methodological approach that could, in principle, be adapted to other WiFi-enabled bio-loggers with similar capabilities. While device-specific implementations would differ, the underlying conceptual framework remains valid.
**3. Does the title accurately reflect that the study evaluates shallow decision-tree–based ML rather than a broader AI framework?**
- We intentionally focused on classical machine learning approaches because they are easier to interpret, computationally lightweight, and allow more explicit analysis of energy expenditure. Decision trees therefore provide a suitable and transparent foundation for investigating the potential of onboard machine learning for energy preservation. More complex approaches could build on this work in future studies.
**4. The abstract claims energy costs are reduced to “a tenth.” Under what behavioral frequency assumptions was this calculated?**
- As described in Section 2.3.5 (“Classification”), our calculations indicate that, under the assumptions used in our comparison, performing one classification operation is approximately one order of magnitude less expensive than transmitting a single sensor reading. These estimates depend strongly on implementation details, overhead considerations, and deployment conditions.
**5. How robust are the reported >80% accuracies given the small sample size (n = 3)?**
- As described, this study represents an initial proof-of-concept phase intended to assess feasibility and theoretical potential. Future studies should include larger datasets and species-specific deployments to evaluate practical performance under field conditions. The controlled human dataset was chosen to provide an interpretable and reproducible experimental setting. Given the fast developments in the field and to facilitate further developments on a larger scale we feel it is important to report on our current status.
**6. Why are no confidence intervals or uncertainty estimates reported for performance metrics?**
- Given the small sample size (n = 3), confidence intervals and uncertainty estimates would not provide meaningful statistical interpretation. We now discuss this limitation more explicitly throughout the manuscript, including in Section 2.4 ("Pilot Data Collection").
**7. Is the statement that gyroscopes are “less commonly utilized” supported by literature evidence?**
- Yes. We added an additional citation in the Introduction section to support the statement regarding the comparatively less common use of gyroscopes in on-board behavior-classification tasks.
**8. What is the precise novelty compared to prior onboard ML work (e.g., Korpela et al.)?**
- We addressed this point by adding the following sentence to the Introduction: "Through those three aspects, we further develop the idea of Korpela et al. by incorporating more sensor types, elaborating on the effective theoretical energy savings, and how those translate into the actual data transmission."
**9. How does this approach differ from embedded TinyML systems such as those implemented using TensorFlow Lite?**
- We did not aim to develop a novel TinyML methodology. Instead, we apply a simple and well-established machine learning approach within the context of animal sensing and evaluate its potential for energy preservation in bio-logging applications.
**10. Why was WiFi selected as the focal communication protocol instead of lower-power alternatives?**
- Since not all bio-logging applications can be achieved using low-power communication protocols alone, WiFi-capable systems have become increasingly relevant. We therefore discuss the advantages and practical motivations for WiFi-based systems in the Introduction section.
**11. Have the authors quantitatively compared their method against compression-only or adaptive duty-cycling strategies?**
- We thank the reviewer for this interesting suggestion. We now address alternative architectural improvements, including adaptive duty cycling and related strategies, in a dedicated Discussion paragraph. The current study remains limited to controlled human experiments as part of the proof-of-concept design.
**12. Why were only three human participants included?**
- We included no more than three participants because the goal was to create a small, controlled demonstration dataset suitable for evaluating the conceptual behavior of the proposed system. The study was not designed as a classifier-optimization benchmark, but rather as a feasibility investigation. Hence, three participants were deemed to be enough.
**13. Was cross-validation (e.g., leave-one-subject-out) performed to test generalization?**
- Yes. We report these experiments in the Results section. We additionally note that future work should include larger animal datasets and more extensive leave-one-subject-out validation approaches to evaluate generalization capabilities more rigorously.
**14. Were training and testing datasets strictly separated to prevent data leakage?**
- We thank the reviewer for identifying this omission. We added the train-test split information (0.3 ratio) to the "Modelling and Inference" section.
**15. How was overfitting assessed, particularly for tree depth 14?**
- Overfitting was assessed through cross-individual validation. Applying the EA60 classifier (trained on participant 1) to ED3C data (participant 3) resulted in only 27.13% accuracy for tree depth 14 and 25.44% accuracy for depth 7. This substantial performance drop suggests that deeper trees learned participant-specific patterns rather than broadly transferable behavioral signatures. We now discuss this limitation more explicitly in the manuscript.
**16. Why were only simple statistical features (mean and variance) used?**
- We deliberately chose simple statistical features (mean and variance) for several reasons: computational efficiency, proof-of-concept simplicity, reduced onboard energy consumption, and interpretability. Future work should investigate more sophisticated feature extraction methods and their associated energy-performance trade-offs.
**17. Were time-domain and frequency-domain features evaluated?**
- No. Only time-domain features were evaluated in this proof-of-concept study. Frequency-domain features such as spectral analysis or FFT-based measures were not included. This limitation was due to our focus on demonstrating basic feasibility with minimal computational overhead for onboard processing. Frequency-domain features could potentially improve classification performance and should be evaluated in future work, particularly for detecting periodic locomotion patterns.
**18. Is the proposed GVeDBA metric physiologically validated?**
- No, GVeDBA is not physiologically validated. We now clearly state in the manuscript that it represents an experimental feature inspired by rotational movement and does not currently constitute an established biological metric.
**19. Why were only decision trees tested?**
- Decision trees were selected because they are interpretable, computationally lightweight, and straightforward to deploy on embedded systems with constrained resources. They therefore align well with the objectives of this proof-of-concept study.
**20. How does performance compare with lightweight ensemble models (e.g., random forest)?**
- We agree that ensemble approaches such as random forests could improve robustness and predictive performance. However, they also introduce additional computational and energy costs, which are important considerations in low-power embedded systems.
**21. How was hyperparameter tuning conducted?**
- Hyperparameter tuning was limited to specifying the maximum tree depth. In practice, the generated trees often became shallower than the defined maximum depth depending on the training data.
**22. Was real onboard power consumption measured experimentally?**
- We thank the reviewer for this important point. Empirical measurement of onboard energy consumption represents an important next step for experimental analyses and should be addressed in future deployment studies.
**23. What is the memory footprint of the deployed model on the MCU?**
- We added the memory footprint of the deployed models to the Results section. On average, decision tree header files required approximately 4.9 kB for depth 7 and 21.5 kB for depth 14, which fits comfortably within the WildFi hardware constraints.
**24. Was classification latency measured under real deployment conditions?**
- Thanks to the reviewer for this important question. Indeed classification latency measurement under deployment conditions poses a relevant future direction for practical studies to our conceptual work.
**25. Why does the energy model ignore WiFi connection and handshake overhead?**
- We agree that WiFi connection overhead cannot be neglected entirely. We therefore expanded our discussion of overhead considerations throughout the manuscript.
**26. Are the reported 99.9903% energy savings realistic under field transmission conditions?**
- No. The 99.9903% value represents an extreme theoretical upper bound rather than a realistic field expectation. We revised the manuscript to emphasize more realistic deployment scenarios and practical savings estimates.
**27. Why is energy reported in mA rather than Joules, and were battery discharge characteristics considered?**
- Thank you for identifying this issue. We corrected the reporting inconsistency and now consistently report overall energy expenditure rather than intermediate sub-calculations.
**28. Why is inter-individual generalization as low as 27%, and what does this imply for deployment?**
- The low inter-individual generalization primarily reflects the intentionally small and highly controlled dataset design. The dataset was not intended to create broadly transferable models, but rather to provide a transparent and interpretable proof-of-concept benchmark.
**29. Were statistical significance tests performed between models?**
- No formal statistical significance testing was performed, as the study focuses on feasibility rather than population-level statistical inference.
**30. Why are uncertainty measures (confidence intervals) absent?**
- Confidence intervals were omitted because the small sample size would not support meaningful statistical interpretation. We now discuss this limitation more prominently.
**31. Human head-mounted motion differs substantially from animal-mounted tags. How transferable are these results?**
- We agree that human head-mounted motion differs substantially from animal-mounted bio-loggers. However, works such as by Korpela et al. indicate feasibility of animal behavior classification. Our contribution lies primarily in the methodological framework and energy analysis rather than direct biological generalization. A future study would require to test the practical deployment on animals.
**32. How would species-specific gait variability affect classifier performance?**
- We acknowledge this important limitation and expanded the Discussion section accordingly. We explicitly address that: (1) animal bio-loggers may be mounted differently (back, limbs), and (2) species-specific validation is required. Species-specific gait variability and mounting location differences will likely influence classifier performance and therefore require dedicated validation in future work.
**33. Have any pilot deployments been conducted on animals to validate applicability?**
- No animal deployments were conducted in this study. The present work was intentionally designed as a controlled proof-of-concept study to establish methodological feasibility before moving toward field validation.
**34. Why are extreme theoretical energy savings emphasized rather than realistic deployment scenarios?**
- We revised the manuscript to emphasize realistic deployment scenarios and practical energy savings while presenting the extreme theoretical estimates only as upper bounds.
**35. Does behavior-based filtering risk discarding rare but ecologically important behaviors?**
- Yes. We now explicitly discuss the possibility that behavior-based filtering could discard rare but ecologically important events and identify this as an important limitation of the proposed approach.
**36. The manuscript is framed as proof-of-concept, yet conclusions are strongly worded. Is the framing proportional to the evidence?**
- We revised the manuscript language throughout to better reflect the proof-of-concept nature of the study.
**37. Is the full firmware source code publicly available?**
- Yes. The WildFi firmware is available through the original publication by Wild et al., while all source code specific to our study is available through our Zenodo repository.
**38. Can the decision trees be exactly regenerated from the shared dataset?**
- Yes, largely. The decision trees can be regenerated from the shared dataset using the provided code and fixed random seeds. Minor variations may happen with equally good splits during tree generation, floating point behaviour of the CPU, or thread scheduling of the OS, but these do not substantially affect the conclusions.
**39. Since human subjects were recorded, was institutional ethical approval obtained?**
- We thank the reviewer for raising this important point. The study was conducted in compliance with institutional regulations, and the corresponding waiver document has been included in the submission. Additionally, we renamed Section 2.4 from "Human-based Experiment" to "Pilot Data Collection" to better reflect the nature of the study.
**40. Was informed consent documented?**
- Yes. All participants were informed about the study and provided verbal and written consent before participation.
**41. Does this study demonstrate a generalizable, field-validated system — or a controlled laboratory proof-of-concept limited to one device and three human subjects?**
- Our study is explicitly a controlled laboratory proof-of-concept limited to one device platform and three human participants. We do not claim to present a fully field-validated or broadly generalizable system. Instead, we provide a methodological and theoretical foundation demonstrating the feasibility of onboard ML-based energy preservation strategies for WiFi-enabled bio-loggers, while clearly identifying field validation and species-specific deployment as essential future work.

We thank the editor and reviewers again for their constructive feedback and valuable suggestions.

Sincerely, 
Wilhelm Kerle-Malcharek 
on behalf of all authors 
Department of Computer and Information Science, University of Konstanz, Konstanz,
Germany 
[wilhelm.kerle@uni-konstanz.de](mailto:wilhelm.kerle@uni-konstanz.de)
