# Supplementary material for: Resource-efficient data transmission for WiFi-capable bio-loggers based on machine learning
Source: PLoS One. 2026 Jul 24;21(7):e0354146. doi: 10.1371/journal.pone.0354146 (PMC13399348; doi:10.1371/journal.pone.0354146)
Supplement: Peer review history

# Peer review: Decision Letter 0

**DOI:** 10.1371/journal.pone.0354146.r001

**Authors:** Zhiyuan Ren

12 Mar 2026

PONE-D-25-67709

Resource-efficient data transmission for WiFi-capable bio-loggers based on machine learning

PLOS One

Dear Dr. Kerle-Malcharek,

Thank you for submitting your manuscript to PLOS ONE. After careful consideration, we feel that it has merit but does not fully meet PLOS ONE’s publication criteria as it currently stands. Therefore, we invite you to submit a revised version of the manuscript that addresses the points raised during the review process.

Please submit your revised manuscript by Apr 26 2026 11:59PM. If you will need more time than this to complete your revisions, please reply to this message or contact the journal office at plosone@plos.org. When you're ready to submit your revision, log on to https://www.editorialmanager.com/pone/ and select the 'Submissions Needing Revision' folder to locate your manuscript file.

Please include the following items when submitting your revised manuscript:

- A letter that responds to each point raised by the academic editor and reviewer(s). You should upload this letter as a separate file labeled 'Response to Reviewers'.
- A marked-up copy of your manuscript that highlights changes made to the original version. You should upload this as a separate file labeled 'Revised Manuscript with Track Changes'.
- An unmarked version of your revised paper without tracked changes. You should upload this as a separate file labeled 'Manuscript'.

If you would like to make changes to your financial disclosure, please include your updated statement in your cover letter. Guidelines for resubmitting your figure files are available below the reviewer comments at the end of this letter.

If applicable, we recommend that you deposit your laboratory protocols in protocols.io to enhance the reproducibility of your results. Protocols.io assigns your protocol its own identifier (DOI) so that it can be cited independently in the future. For instructions see: https://journals.plos.org/plosone/s/submission-guidelines#loc-laboratory-protocols. Additionally, PLOS ONE offers an option for publishing peer-reviewed Lab Protocol articles, which describe protocols hosted on protocols.io. Read more information on sharing protocols at https://plos.org/protocols?utm_medium=editorial-email&utm_source=authorletters&utm_campaign=protocols.

We look forward to receiving your revised manuscript.

Kind regards,

Zhiyuan Ren

Academic Editor

PLOS One

Journal Requirements:

When submitting your revision, we need you to address these additional requirements.

1. Please ensure that your manuscript meets PLOS ONE's style requirements, including those for file naming. The PLOS ONE style templates can be found at

https://journals.plos.org/plosone/s/file?id=wjVg/PLOSOne_formatting_sample_main_body.pdf and

https://journals.plos.org/plosone/s/file?id=ba62/PLOSOne_formatting_sample_title_authors_affiliations.pdf

2. Please note that PLOS One has specific guidelines on code sharing for submissions in which author-generated code underpins the findings in the manuscript. In these cases, we expect all author-generated code to be made available without restrictions upon publication of the work. Please review our guidelines at https://journals.plos.org/plosone/s/materials-and-software-sharing#loc-sharing-code and ensure that your code is shared in a way that follows best practice and facilitates reproducibility and reuse.

3. Thank you for stating the following in the Acknowledgments Section of your manuscript:

“We acknowledge funding by DFG, under Germany’s Excellence Strategy – EXC 2117 – 422037984, and DFG project ID 251654672 – TRR 161. Furthermore, the authors thank the Max-Planck-Institute of Animal Behaviour for their support. We are grateful for funding support from the Academy for the Protection of Zoo Animals and Wildlife e.V. This research is funded in part by the Gordon and Betty Moore Foundation through grant GBMF10539.”

We note that you have provided funding information that is not currently declared in your Funding Statement. However, funding information should not appear in the Acknowledgments section or other areas of your manuscript. We will only publish funding information present in the Funding Statement section of the online submission form.

Please remove any funding-related text from the manuscript and let us know how you would like to update your Funding Statement. Currently, your Funding Statement reads as follows:

“This research is funded in part by the Gordon and Betty Moore Foundation through grant GBMF1053. The main author is funded by DFG, under Germany’s Excellence Strategy – EXC 2117 – 422037984, and DFG project ID 251654672 – TRR 161.”

Please include your amended statements within your cover letter; we will change the online submission form on your behalf.

4. We note that the grant information you provided in the ‘Funding Information’ and ‘Financial Disclosure’ sections do not match.

When you resubmit, please ensure that you provide the correct grant numbers for the awards you received for your study in the ‘Funding Information’ section.

5. Thank you for stating the following financial disclosure:

“This research is funded in part by the Gordon and Betty Moore Foundation through grant GBMF1053. The main author is funded by DFG, under Germany’s Excellence Strategy – EXC 2117 – 422037984, and DFG project ID 251654672 – TRR 161.”

Please state what role the funders took in the study.  If the funders had no role, please state: "The funders had no role in study design, data collection and analysis, decision to publish, or preparation of the manuscript."

If this statement is not correct you must amend it as needed.

Please include this amended Role of Funder statement in your cover letter; we will change the online submission form on your behalf.

6. We note that Figure 1 in your submission contain copyrighted images. All PLOS content is published under the Creative Commons Attribution License (CC BY 4.0), which means that the manuscript, images, and Supporting Information files will be freely available online, and any third party is permitted to access, download, copy, distribute, and use these materials in any way, even commercially, with proper attribution. For more information, see our copyright guidelines: http://journals.plos.org/plosone/s/licenses-and-copyright.

We require you to either (1) present written permission from the copyright holder to publish these figures specifically under the CC BY 4.0 license, or (2) remove the figures from your submission:

1. You may seek permission from the original copyright holder of Figure(s) [#] to publish the content specifically under the CC BY 4.0 license.

We recommend that you contact the original copyright holder with the Content Permission Form (http://journals.plos.org/plosone/s/file?id=7c09/content-permission-form.pdf) and the following text:

“I request permission for the open-access journal PLOS ONE to publish XXX under the Creative Commons Attribution License (CCAL) CC BY 4.0 (http://creativecommons.org/licenses/by/4.0/). Please be aware that this license allows unrestricted use and distribution, even commercially, by third parties. Please reply and provide explicit written permission to publish XXX under a CC BY license and complete the attached form.”

Please upload the completed Content Permission Form or other proof of granted permissions as an "Other" file with your submission.

In the figure caption of the copyrighted figure, please include the following text: “Reprinted from [ref] under a CC BY license, with permission from [name of publisher], original copyright [original copyright year].”

2. If you are unable to obtain permission from the original copyright holder to publish these figures under the CC BY 4.0 license or if the copyright holder’s requirements are incompatible with the CC BY 4.0 license, please either i) remove the figure or ii) supply a replacement figure that complies with the CC BY 4.0 license. Please check copyright information on all replacement figures and update the figure caption with source information. If applicable, please specify in the figure caption text when a figure is similar but not identical to the original image and is therefore for illustrative purposes only.

7. If the reviewer comments include a recommendation to cite specific previously published works, please review and evaluate these publications to determine whether they are relevant and should be cited. There is no requirement to cite these works unless the editor has indicated otherwise.

[Note: HTML markup is below. Please do not edit.]

Reviewers' comments:

Reviewer's Responses to Questions

**Comments to the Author**

1. Is the manuscript technically sound, and do the data support the conclusions?

The manuscript must describe a technically sound piece of scientific research with data that supports the conclusions. Experiments must have been conducted rigorously, with appropriate controls, replication, and sample sizes. The conclusions must be drawn appropriately based on the data presented.

Reviewer #1: Yes

Reviewer #2: Partly

**********

2. Has the statistical analysis been performed appropriately and rigorously?

Reviewer #1: Yes

Reviewer #2: No

**********

3. Have the authors made all data underlying the findings in their manuscript fully available?

The PLOS Data policy requires authors to make all data underlying the findings described in their manuscript fully available without restriction, with rare exception (please refer to the Data Availability Statement in the manuscript PDF file). The data should be provided as part of the manuscript or its supporting information, or deposited to a public repository. For example, in addition to summary statistics, the data points behind means, medians and variance measures should be available. If there are restrictions on publicly sharing data—e.g. participant privacy or use of data from a third party—those must be specified.

Reviewer #1: Yes

Reviewer #2: Yes

**********

4. Is the manuscript presented in an intelligible fashion and written in standard English?

PLOS ONE does not copyedit accepted manuscripts, so the language in submitted articles must be clear, correct, and unambiguous. Any typographical or grammatical errors should be corrected at revision, so please note any specific errors here.

Reviewer #1: Yes

Reviewer #2: Yes

**********

5. Review Comments to the Author

Please use the space provided to explain your answers to the questions above. You may also include additional comments for the author, including concerns about dual publication, research ethics, or publication ethics. (Please upload your review as an attachment if it exceeds 20,000 characters)

Reviewer #1: 1) The manuscript focuses heavily on device-level energy savings through data filtering but does not adequately address network-level energy optimization strategies that could complement the proposed approach. While the authors mention IoT strategies briefly, the discussion remains primarily at the individual logger level. Consider incorporating findings from [Xu et al. (2022): Energy Consumption Minimization for Data Collection From Wirelessly-Powered IoT Sensors: Session-Specific Optimal Design With DRL. IEEE Sensors Journal, 22(20), 19886-19896. doi: 10.1109/JSEN.2022.3205017] which presents energy consumption minimization techniques for data collection from wirelessly-powered IoT sensors. Their session-specific optimal design using deep reinforcement learning could provide valuable context for optimizing when and how bio-loggers transmit data, especially in multi-device deployment scenarios common in animal behavior studies. Add this discussion in Section 2.2 (General Concepts) or the Discussion section when addressing transmission overhead considerations.

2. The manuscript acknowledges WiFi's higher energy cost compared to lightweight protocols but does not explore potential improvements at the communication protocol level that could further reduce energy consumption while maintaining WiFi's advantages. At Section 2.2 (General Concepts) or Section 5 (Discussion) when addressing transmission overhead and connection time considerations, add this statement: The work by [Liu et al. (2022): Concurrent Low-Power Listening: A New Design Paradigm for Duty-Cycling Communication. ACM Trans. Sen. Netw., 19(1). doi: 10.1145/3517013] on Concurrent Low-Power Listening presents a new design paradigm for duty-cycling communication that could be highly relevant. Their approach demonstrates how duty-cycling can be optimized to reduce energy consumption without sacrificing network performance. This could inform future iterations of the WildFi tag's communication strategy.

3. The authors note that classifiers trained on one individual perform poorly on others (e.g., EA60 classifier on ED3C yielded only 27.13% accuracy). This limitation is acknowledged but not adequately addressed in terms of potential solutions. Recent advances in transfer learning could offer pathways to address this limitation. At Section 5 (Discussion) when addressing inter-individual classifier performance limitations. consider citing [Liu et al. (2025): Establishing a Digital Twin Diagnostic Model Based on Cross-Device Transfer Learning. IEEE Transactions on Instrumentation and Measurement, 74, 1-10. doi: 10.1109/TIM.2025.3562973] which presents a digital twin diagnostic model based on cross-device transfer learning. Their approach demonstrates how models can be adapted across different devices or individuals, potentially enabling more generalizable behavior classifiers. Additionally, [Yan et al. (2025): A Generalizable Load Recognition Method in NILM Based on Transferable Random Forest. IEEE Transactions on Instrumentation and Measurement, 74. doi: 10.1109/TIM.2025.3570355] on transferable random forests for load recognition could provide insights specific to tree-based models like those used in this study.

4. The manuscript treats behavior classification as a static problem, but animal behaviors often exhibit temporal patterns and may change over time due to environmental factors, habituation, or physiological states. At Section 3.3 (Feature Calculation) or Section 5 (Discussion) when addressing the complexity of animal behavior patterns, consider adding: Two recent papers could strengthen this aspect. First, [Wu et al. (2025): Tidal-Like Concept Drift in RIS-Covered Buildings: When Programmable Wireless Environments Meet Human Behaviors. IEEE Wireless Communications, 1-8. doi: 10.1109/MWC.2025.3600792] discusses concept drift in programmable wireless environments when human behaviors change - a relevant consideration for long-term animal behavior studies where behavioral patterns may shift. Second, [Luo et al. (2025): A2Tformer: Addressing Temporal Bias and Non-Stationarity in Transformer-Based IoT Time Series Classification. IEEE Internet of Things Journal, 1. doi: 10.1109/JIOT.2025.3595765] addresses temporal bias and non-stationarity in transformer-based IoT time series classification, offering methodological insights that could be adapted for bio-logging applications.

5. The manuscript focuses on single-device energy savings but does not address scenarios where multiple bio-loggers operate in proximity (e.g., studying social animals in groups). In such cases, network-level coordination could yield additional energy savings. The work by [Cao et al. (2021): RFID Reader Anticollision Based on Distributed Parallel Particle Swarm Optimization. IEEE internet of things journal, 8(5), 3099-3107. doi: 10.1109/JIOT.2020.3033473] on RFID reader anticollision using distributed parallel particle swarm optimization demonstrates how multi-device interference can be managed. While their context differs, the principles of coordinating multiple transmitting devices could inform future extensions of this work for group-deployment scenarios.

6. The manuscript mentions on-board classification but does not discuss the broader ecosystem of edge computing that could support more sophisticated processing while maintaining energy efficiency. At Section 2.2 (General Concepts) when discussing transmission protocols, or Section 5 (Discussion) as a future direction. Consider adding [ Xu et al. (2025): MPAEE: A Multipath Adaptive Energy-Efficient Routing Scheme for Low Earth Orbit-Based Industrial Internet of Things. IEEE Internet of Things Journal, 12(17), 34793-34805. doi: 10.1109/JIOT.2025.3581314] presents a multipath adaptive energy-efficient routing scheme for low Earth orbit-based industrial IoT. While specifically focused on satellite communication, their approach to adaptive, energy-aware routing could inspire similar strategies for bio-logging data transmission, particularly for loggers that may need to route data through intermediate nodes.

7. The authors demonstrate the effectiveness of decision trees but do not compare their approach with other machine learning methods that might offer better trade-offs for specific behaviors or sensor configurations. When addressing limitations of decision trees compared to more complex models, consider citing [Jin et al. (2022): A fixed-time convergent and noise-tolerant zeroing neural network for online solution of time-varying matrix inversion. Applied Soft Computing, 130, 109691. doi: https://doi.org/10.1016/j.asoc.2022.109691] which presents a fixed-time convergent zeroing neural network for online solutions. While their application differs, their approach to time-varying system modeling could inspire more sophisticated on-board processing techniques that adapt to changing animal behaviors over time. This could complement the decision tree approach presented here.

8. The energy calculations in Section 2.2 are helpful but the assumptions could be stated more explicitly. The authors assume invariant transmission rate and neglect overheads, which is reasonable for initial estimates, but the limitations of these assumptions should be discussed more prominently.

9. The finding about gyroscope importance (appearing in top-performing feature combinations) is valuable. The authors provide a plausible explanation (head movement during standing vs. running). This could be expanded with more biomechanical reasoning, possibly citing literature on head stabilization during locomotion in different species.

10. The controlled human experiment provides a useful proof-of-concept, but the manuscript would benefit from a more detailed discussion of challenges specific to animal deployment: weather effects on sensors, attachment stability over time, and behavioral variability within and across species.

Reviewer #2: This manuscript presents an interesting proof-of-concept approach for reducing WiFi transmission energy in bio-loggers using onboard machine learning. The topic is timely and relevant for long-term wildlife monitoring. However, the study is based solely on controlled human data (n=3), which limits statistical robustness and raises concerns about generalizability to animal-borne systems. Inter-individual transfer performance is low, and cross-validation procedures are not sufficiently detailed. Energy savings are modeled theoretically, without empirical onboard power measurements, and important transmission overheads are not incorporated. The reported energy reductions therefore appear optimistic. The novelty relative to prior embedded ML work is not clearly articulated. Additionally, the risk of losing rare but ecologically important behaviors through filtering is not fully addressed. Clarification regarding ethical approval for human participants is also needed. Substantial revisions are required to strengthen methodological rigor and ecological relevance.

**********

6. PLOS authors have the option to publish the peer review history of their article (what does this mean?). If published, this will include your full peer review and any attached files.

If you choose “no”, your identity will remain anonymous but your review may still be made public.

**Do you want your identity to be public for this peer review?**  For information about this choice, including consent withdrawal, please see our Privacy Policy.

Reviewer #1: No

Reviewer #2: No

**********

[NOTE: If reviewer comments were submitted as an attachment file, they will be attached to this email and accessible via the submission site. Please log into your account, locate the manuscript record, and check for the action link "View Attachments". If this link does not appear, there are no attachment files.]

To ensure your figures meet our technical requirements, please review our figure guidelines: https://journals.plos.org/plosone/s/figures

You may also use PLOS’s free figure tool, NAAS, to help you prepare publication quality figures: https://journals.plos.org/plosone/s/figures#loc-tools-for-figure-preparation.

NAAS will assess whether your figures meet our technical requirements by comparing each figure against our figure specifications.

Attachment Submitted filename: renamed_fdb09.docx

# Author comment: Author response to Decision Letter 1

**DOI:** 10.1371/journal.pone.0354146.r002

16 May 2026

Dear esteemed Colleagues,

Thank you very much for the opportunity to revise and resubmit our manuscript to PLOS ONE. We appreciate the constructive feedback provided by the editor and reviewers. Their comments helped us improve the clarity, rigor, and framing of our work substantially.

With respect to the comments, we would like to highlight the following changes and clarifications.

Editing issues

1. Please ensure that your manuscript meets PLOS ONE's style requirements, including those for file naming.

- We adapted the manuscript to match the PLOS ONE style templates to the best of our knowledge. This includes, but is not limited to, adapting the figure captions, reformatting the figures, and shortening the abstract to comply with the 300-word restriction. Please let us know if any additional formatting adjustments are required, and we will address them accordingly.

2. Please note that PLOS One has specific guidelines on code sharing for submissions in which author-generated code underpins the findings in the manuscript. In these cases, we expect all author-generated code to be made available without restrictions upon publication of the work.

- Regarding code sharing, we improved our Zenodo repository to facilitate reproducibility. The updated DOI has already been incorporated into the manuscript. Specifically, we cleaned up legacy code components and added a README file to improve usability and reproducibility.

3. We note that you have provided funding information that is not currently declared in your Funding Statement. However, funding information should not appear in the Acknowledgments section or other areas of your manuscript. We will only publish funding information present in the Funding Statement section of the online submission form.

- The detailed funding information has been removed from the Acknowledgements section, in accordance with the journal guidelines, and we understand that it will instead be included in the appropriate funding section during production.

4. We note that the grant information you provided in the ‘Funding Information’ and ‘Financial Disclosure’ sections do not match.

- Thank you very much for pointing this out. The correct funding information is 1: DFG, project ID 251654672--TRR 161; 2: Academy for the Protection of Zoo Animals and Wildlife e.V and the Gordon and Betty Moore Foundation grant number GBMF10539. We will ensure that this information is correctly reflected during resubmission.

For the Acknowledgments section, the following funding statement is the correct one:

“We acknowledge funding by DFG, under Germany’s Excellence Strategy – EXC 2117 – 422037984, and DFG project ID 251654672 – TRR 161. Furthermore, we acknowledge funding support from the Academy for the Protection of Zoo Animals and Wildlife e.V., and the Gordon and Betty Moore Foundation through grant GBMF10539.”

5. Please state what role the funders took in the study.  If the funders had no role, please state: "The funders had no role in study design, data collection and analysis, decision to publish, or preparation of the manuscript."

- Thank you for identifying this issue. We added the proposed statement: "The funders had no role in study design, data collection and analysis, decision to publish, or preparation of the manuscript."

6. We require you to either (1) present written permission from the copyright holder to publish these figures specifically under the CC BY 4.0 license, or (2) remove the figures from your submission

- Permission for Figure 1 has been granted by the copyright holder, and we uploaded the corresponding permission form. We also added the required note to the figure caption.

Reviewer #1

1. The manuscript focuses heavily on device-level energy savings through data filtering but does not adequately address network-level energy optimization strategies that could complement the proposed approach. While the authors mention IoT strategies briefly, the discussion remains primarily at the individual logger level. Consider incorporating findings from [Xu et al. (2022): Energy Consumption Minimization for Data Collection From Wirelessly-Powered IoT Sensors: Session-Specific Optimal Design With DRL. IEEE Sensors Journal, 22(20), 19886-19896. doi: 10.1109/JSEN.2022.3205017] which presents energy consumption minimization techniques for data collection from wirelessly-powered IoT sensors. Their session-specific optimal design using deep reinforcement learning could provide valuable context for optimizing when and how bio-loggers transmit data, especially in multi-device deployment scenarios common in animal behavior studies. Add this discussion in Section 2.2 (General Concepts) or the Discussion section when addressing transmission overhead considerations.

- We thank the reviewer for this valuable suggestion. While network-level optimization is beyond the scope of our proof-of-concept study, which primarily focuses on device-level energy consumption minimization, we agree that adaptive transmission strategies are highly relevant for broader deployment scenarios. We therefore added a dedicated paragraph in the Discussion section addressing future improvements in this direction.

2. The manuscript acknowledges WiFi's higher energy cost compared to lightweight protocols but does not explore potential improvements at the communication protocol level that could further reduce energy consumption while maintaining WiFi's advantages. At Section 2.2 (General Concepts) or Section 5 (Discussion) when addressing transmission overhead and connection time considerations, add this statement: The work by [Liu et al. (2022): Concurrent Low-Power Listening: A New Design Paradigm for Duty-Cycling Communication. ACM Trans. Sen. Netw., 19(1). doi: 10.1145/3517013] on Concurrent Low-Power Listening presents a new design paradigm for duty-cycling communication that could be highly relevant. Their approach demonstrates how duty-cycling can be optimized to reduce energy consumption without sacrificing network performance. This could inform future iterations of the WildFi tag's communication strategy.

- We thank the reviewer for this helpful input. Bio-loggers are frequently deployed on multiple individuals in close proximity. Since WiFi-capable loggers already commonly rely on gateways to reduce WiFi connection costs, considerations regarding interference avoidance are highly relevant. We therefore incorporated this aspect into the Discussion section.

3. The authors note that classifiers trained on one individual perform poorly on others (e.g., EA60 classifier on ED3C yielded only 27.13% accuracy). This limitation is acknowledged but not adequately addressed in terms of potential solutions. Recent advances in transfer learning could offer pathways to address this limitation. At Section 5 (Discussion) when addressing inter-individual classifier performance limitations. consider citing [Liu et al. (2025): Establishing a Digital Twin Diagnostic Model Based on Cross-Device Transfer Learning. IEEE Transactions on Instrumentation and Measurement, 74, 1-10. doi: 10.1109/TIM.2025.3562973] which presents a digital twin diagnostic model based on cross-device transfer learning. Their approach demonstrates how models can be adapted across different devices or individuals, potentially enabling more generalizable behavior classifiers. Additionally, [Yan et al. (2025): A Generalizable Load Recognition Method in NILM Based on Transferable Random Forest. IEEE Transactions on Instrumentation and Measurement, 74. doi: 10.1109/TIM.2025.3570355] on transferable random forests for load recognition could provide insights specific to tree-based models like those used in this study.

- We appreciate the reviewer’s suggestion regarding transfer learning approaches for improving inter-individual performance. We added a paragraph in the Discussion section addressing transfer learning using decision trees. While the suggested publications raise important points, approaches such as random forests introduce additional computational and energy complexity for onboard deployment. A detailed exploration of these methods would extend beyond the scope of the present proof-of-concept study.

4. The manuscript treats behavior classification as a static problem, but animal behaviors often exhibit temporal patterns and may change over time due to environmental factors, habituation, or physiological states. At Section 3.3 (Feature Calculation) or Section 5 (Discussion) when addressing the complexity of animal behavior patterns, consider adding: Two recent papers could strengthen this aspect. First, [Wu et al. (2025): Tidal-Like Concept Drift in RIS-Covered Buildings: When Programmable Wireless Environments Meet Human Behaviors. IEEE Wireless Communications, 1-8. doi: 10.1109/MWC.2025.3600792] discusses concept drift in programmable wireless environments when human behaviors change - a relevant consideration for long-term animal behavior studies where behavioral patterns may shift. Second, [Luo et al. (2025): A2Tformer: Addressing Temporal Bias and Non-Stationarity in Transformer-Based IoT Time Series Classification. IEEE Internet of Things Journal, 1. doi: 10.1109/JIOT.2025.3595765] addresses temporal bias and non-stationarity in transformer-based IoT time series classification, offering methodological insights that could be adapted for bio-logging applications.

- We thank the reviewer for this valuable input. We agree that concept drift can be highly relevant in this context, particularly for long-term deployments such as studies on migratory animals. While the first proposed work focuses primarily on indoor applications and therefore aligns less directly with our use case, the work by Luo et al. fits well within our discussion and has been incorporated accordingly.

5. The manuscript focuses on single-device energy savings but does not address scenarios where multiple bio-loggers operate in proximity (e.g., studying social animals in groups). In such cases, network-level coordination could yield additional energy savings. The work by [Cao et al. (2021): RFID Reader Anticollision Based on Distributed Parallel Particle Swarm Optimization. IEEE internet of things journal, 8(5), 3099-3107. doi: 10.1109/JIOT.2020.3033473] on RFID reader anticollision using distributed parallel particle swarm optimization demonstrates how multi-device interference can be managed. While their context differs, the principles of coordinating multiple transmitting devices could inform future extensions of this work for group-deployment scenarios.

- We agree that this work is relevant and have added it to the Discussion section.

6. The manuscript mentions on-board classification but does not discuss the broader ecosystem of edge computing that could support more sophisticated processing while maintaining energy efficiency. At Section 2.2 (General Concepts) when discussing transmission protocols, or Section 5 (Discussion) as a future direction. Consider adding [ Xu et al. (2025): MPAEE: A Multipath Adaptive Energy-Efficient Routing Scheme for Low Earth Orbit-Based Industrial Internet of Things. IEEE Internet of Things Journal, 12(17), 34793-34805. doi: 10.1109/JIOT.2025.3581314] presents a multipath adaptive energy-efficient routing scheme for low Earth orbit-based industrial IoT. While specifically focused on satellite communication, their approach to adaptive, energy-aware routing could inspire similar strategies for bio-logging data transmission, particularly for loggers that may need to route data through intermediate nodes.

- We agree that this work is relevant in the context of broader energy-preservation ecosystems. However, it is less directly applicable to WildFi bio-loggers specifically, since WiFi-capable bio-loggers typically rely on gateways to avoid routing overhead, which itself can be energetically expensive. We added a short elaboration on this point in the “General Concepts” section as part of our extended discussion of overhead considerations.

7. The authors demonstrate the effectiveness of decision trees but do not compare their approach with other machine learning methods that might offer better trade-offs for specific behaviors or sensor configurations. When addressing limitations of decision trees compared to more complex models, consider citing [Jin et al. (2022): A fixed-time convergent and noise-tolerant zeroing neural network for online solution of time-varying matrix inversion. Applied Soft Computing, 130, 109691. doi: https://doi.org/10.1016/j.asoc.2022.109691] which presents a fixed-time convergent zeroing neural network for online solutions. While their application differs, their approach to time-varying system modeling could inspire more sophisticated on-board processing techniques that adapt to changing animal behaviors over time. This could complement the decision tree approach presented here.

- We thank the reviewer for this suggestion. We already discussed alternative approaches briefly in the Discussion section; however, we have now expanded this part and incorporated the proposed work accordingly.

8. The energy calculations in Section 2.2 are helpful but the assumptions could be stated more explicitly. The authors assume invariant transmission rate and neglect overheads, which is reasonable for initial estimates, but the limitations of these assumptions should be discussed more prominently.

- We thank the reviewer for this important remark. We addressed this point in greater detail in the "General Concepts" section, where we discuss potential distortions, possible transmission costs, and overhead assumptions more explicitly.

9. The finding about gyroscope importance (appearing in top-performing feature combinations) is valuable. The authors provide a plausible explanation (head movement during standing vs. running). This could be expanded with more biomechanical reasoning, possibly citing literature on head stabilization during locomotion in different species.

- We thank the reviewer for this input and provide two works on head stabilization in context - humans and macaques - to show that even between species that are morphologically close to each other significant differences that can be captured with gyroscopes can be observed. Please find the corresponding passage in the Discussion.

10. The controlled human experiment provides a useful proof-of-concept, but the manuscript would benefit from a more detailed discussion of challenges specific to animal deployment: weather effects on sensors, attachment stability over time, and behavioral variability within and across species.

- We are grateful for the reviewer’s comment and agree that a more detailed discussion of deployment-related challenges strengthens the manuscript’s perspective on real-world applicability.

Reviewer #2)

We thank Reviewer #2 for their extensive input on our manuscript. We would like to emphasize again the proof-of-concept nature of the present study. The controlled human dataset (n = 3) was not intended to establish a highly generalizable behavioral classifier, but rather to provide a controlled and interpretable dataset that allows us to investigate the conceptual relationship between onboard classification and energy preservation.

We fully agree that the statistical robustness of the presented results is limited by the small sample size, and we have revised the manuscript to emphasize this point more clearly throughout.

Our primary aim was not classifier optimization, but rather the investigation of energy-saving potentials. The extensive discussion of classifier structure and decision trees was included to provide sufficient methodological context for interpreting the energy-saving considerations under practical deployment assumptions.

We also agree that transmission overheads should not be omitted entirely, even in a proof-of-concept study. We therefore expanded the discussion of overheads substantially throughout the manuscript.

Similarly, we now state more clearly that the reported energy reductions represent theoretical rather than practical savings estimates. We additionally clarified the novelty of our contribution, expanded the discussion regarding potentially missed behaviors due to filtering, and added clarification regarding ethical approval.

1.

Attachment Submitted filename: Response to Reviewers.docx

# Peer review: Decision Letter 1

**DOI:** 10.1371/journal.pone.0354146.r003

**Authors:** Zhiyuan Ren

5 Jul 2026

Resource-efficient data transmission for WiFi-capable bio-loggers based on machine learning

PONE-D-25-67709R1

Dear Dr. Kerle-Malcharek,

We’re pleased to inform you that your manuscript has been judged scientifically suitable for publication and will be formally accepted for publication once it meets all outstanding technical requirements.

Within one week, you’ll receive an e-mail detailing the required amendments. When these have been addressed, you’ll receive a formal acceptance letter and your manuscript will be scheduled for publication.

An invoice will be generated when your article is formally accepted. Please note, if your institution has a publishing partnership with PLOS and your article meets the relevant criteria, all or part of your publication costs will be covered. Please make sure your user information is up-to-date by logging into Editorial Manager at Editorial Manager® and clicking the ‘Update My Information' link at the top of the page. For questions related to billing, please contact billing support.

If your institution or institutions have a press office, please notify them about your upcoming paper to help maximize its impact. If they’ll be preparing press materials, please inform our press team as soon as possible -- no later than 48 hours after receiving the formal acceptance. Your manuscript will remain under strict press embargo until 2 pm Eastern Time on the date of publication. For more information, please contact onepress@plos.org.

Kind regards,

Zhiyuan Ren

Academic Editor

PLOS One

Additional Editor Comments (optional):

Reviewers' comments:

Reviewer's Responses to Questions

**Comments to the Author**

1. If the authors have adequately addressed your comments raised in a previous round of review and you feel that this manuscript is now acceptable for publication, you may indicate that here to bypass the “Comments to the Author” section, enter your conflict of interest statement in the “Confidential to Editor” section, and submit your "Accept" recommendation.

Reviewer #1: (No Response)

Reviewer #3: All comments have been addressed

**********

2. Is the manuscript technically sound, and do the data support the conclusions?

The manuscript must describe a technically sound piece of scientific research with data that supports the conclusions. Experiments must have been conducted rigorously, with appropriate controls, replication, and sample sizes. The conclusions must be drawn appropriately based on the data presented.

Reviewer #1: Yes

Reviewer #3: Yes

**********

3. Has the statistical analysis been performed appropriately and rigorously?

Reviewer #1: Yes

Reviewer #3: Yes

**********

4. Have the authors made all data underlying the findings in their manuscript fully available?

The PLOS Data policy requires authors to make all data underlying the findings described in their manuscript fully available without restriction, with rare exception (please refer to the Data Availability Statement in the manuscript PDF file). The data should be provided as part of the manuscript or its supporting information, or deposited to a public repository. For example, in addition to summary statistics, the data points behind means, medians and variance measures should be available. If there are restrictions on publicly sharing data—e.g. participant privacy or use of data from a third party—those must be specified.

Reviewer #1: Yes

Reviewer #3: Yes

**********

5. Is the manuscript presented in an intelligible fashion and written in standard English?

PLOS ONE does not copyedit accepted manuscripts, so the language in submitted articles must be clear, correct, and unambiguous. Any typographical or grammatical errors should be corrected at revision, so please note any specific errors here.

Reviewer #1: Yes

Reviewer #3: Yes

**********

6. Review Comments to the Author

Please use the space provided to explain your answers to the questions above. You may also include additional comments for the author, including concerns about dual publication, research ethics, or publication ethics. (Please upload your review as an attachment if it exceeds 20,000 characters)

Reviewer #1: The revised version now looks standard and worth publishing in the journal. I recommend the paper for Acceptance

Reviewer #3: The authors have substantially revised the manuscript and have adequately addressed the concerns raised in the previous round of review. In particular, they have clarified the proof-of-concept nature of the study, discussed the limitations related to the small sample size, limited inter-individual generalization, theoretical energy estimates, and future animal-specific validation, and added relevant discussion on transmission overheads, multi-device deployment, transfer learning, concept drift, and practical deployment challenges.

I agree that the paper should be published.

**********

7. PLOS authors have the option to publish the peer review history of their article (what does this mean?). If published, this will include your full peer review and any attached files.

If you choose “no”, your identity will remain anonymous but your review may still be made public.

**Do you want your identity to be public for this peer review?**  For information about this choice, including consent withdrawal, please see our Privacy Policy.

Reviewer #1: **Yes:** Dr. Mukhtar Iderawumi Abdulraheem

Reviewer #3: No

**********

# Editor report: Acceptance letter

**DOI:** 10.1371/journal.pone.0354146.r004

**Authors:** Zhiyuan Ren

PONE-D-25-67709R1

PLOS One

Dear Dr. Kerle-Malcharek,

I'm pleased to inform you that your manuscript has been deemed suitable for publication in PLOS One. Congratulations! Your manuscript is now being handed over to our production team.

At this stage, our production department will prepare your paper for publication. This includes ensuring the following:

* All references, tables, and figures are properly cited

* All relevant supporting information is included in the manuscript submission,

* There are no issues that prevent the paper from being properly typeset

You will receive further instructions from the production team, including instructions on how to review your proof when it is ready. Please keep in mind that we are working through a large volume of accepted articles, so please give us a few days to review your paper and let you know the next and final steps.

Lastly, if your institution or institutions have a press office, please let them know about your upcoming paper now to help maximize its impact. If they'll be preparing press materials, please inform our press team within the next 48 hours. Your manuscript will remain under strict press embargo until 2 pm Eastern Time on the date of publication. For more information, please contact onepress@plos.org.

You will receive an invoice from PLOS for your publication fee after your manuscript has reached the completed accept phase. If you receive an email requesting payment before acceptance or for any other service, this may be a phishing scheme. Learn how to identify phishing emails and protect your accounts at https://explore.plos.org/phishing.

If we can help with anything else, please email us at customercare@plos.org.

Thank you for submitting your work to PLOS ONE and supporting open access.

Kind regards,

PLOS ONE Editorial Office Staff

on behalf of

Professor Zhiyuan Ren

Academic Editor

PLOS One
